# Diversity, community structure, and quantity of eukaryotic phytoplankton revealed using 18S rRNA and plastid 16S rRNA genes and pigment markers: a case study of the Pearl River Estuary

**DOI:** 10.1007/s42995-023-00186-x

**Published:** 2023-07-29

**Authors:** Shumin Xu, Guihao Li, Cui He, Yi Huang, Dan Yu, Huiwen Deng, Zhuyin Tong, Yichong Wang, Christine Dupuy, Bangqin Huang, Zhuo Shen, Jie Xu, Jun Gong

**Affiliations:** 1 School of Marine Sciences, Sun Yat-Sen University (Zhuhai Campus), and Southern Marine Science and Engineering Guangdong Laboratory (Zhuhai), Zhuhai, 519000 China; 2 Guangdong Provincial Key Laboratory of Marine Resources and Coastal Engineering, Guangzhou, 510006 China; 3 State Key Laboratory of Marine Environmental Science, Xiamen University, Xiamen, 361102 China; 4 BIOFEEL, UMRi LIENSs, La Rochelle Université/CNRS, La Rochelle, France; 5 Centre for Regional Oceans, Department of Civil and Environmental Engineering, Faculty of Science and Technology, University of Macau, Macau, China

**Keywords:** Accessory pigments, Chloroplast, Marker gene, Metabarcoding, Phytoplankton

## Abstract

Understanding consistencies and discrepancies in characterizing diversity and quantity of phytoplankton is essential for better modeling ecosystem change. In this study, eukaryotic phytoplankton in the Pearl River Estuary, South China Sea were investigated using nuclear 18S rRNA and plastid 16S or 23S rRNA genes and pigment analysis. It was found that 18S abundance poorly explained the variations in total chlorophyll *a* (Chl-*a*). However, the ratios of log-transformed 18S abundance to Chl-*a* in the major phytoplankton groups were generally environment dependent, suggesting that the ratio has potential as an indicator of the physiological state of phytoplankton. The richness of 18S-based operational taxonomic units was positively correlated with the richness of 16S-based amplicon sequence variants of the whole phytoplankton community, but insignificant or weak for individual phytoplankton groups. Overall, the 18S based, rather than the 16S based, community structure had a greater similarity to pigment-based estimations. Relative to the pigment data, the proportion of haptophytes in the 18S dataset, and diatoms and cryptophytes in the 16S dataset, were underestimated. This study highlights that 18S metabarcoding tends to reflect biomass-based community organization of eukaryotic phytoplankton. Because there were lower copy numbers of plastid 16S than 18S per genome, metabarcoding of 16S probably approximates cell abundance-based community organization. Changes in biomass organization of the pigment-based community were sensitive to environmental changes. Taken together, multiple methodologies are recommended to be applied to more accurately profile the diversity and community composition of phytoplankton in natural ecosystems.

**Supplementary Information:**

The online version contains supplementary material available at 10.1007/s42995-023-00186-x.

## Introduction

Phytoplankton are photosynthetic microbes comprising taxonomically diverse species of cyanobacteria and eukaryotic microalgae in aquatic ecosystems. Marine phytoplankton are responsible for about half of our planet’s annual net primary production (Falkowski et al. 2004; Field et al. 1998). The tremendous importance of phytoplankton in ecology and biogeochemical processes (e.g., cycling of nitrogen, phosphorus, silicate and iron, driving carbon export to deep waters) has triggered the development of chemical and molecular methodologies to characterize their diversity and community composition across time and space (Abaychi and Riley 1979; Chen et al. 2022; Gao et al. 2020; Maki et al. 2017; Treusch et al. 2012; Xie et al. 2022). Relative to classical methodologies based on morphological identification and enumeration (Huang et al. 2021), chemotaxonomy of accessory pigments by high-performance liquid chromatography (HPLC) enables the rapid quantification of major groups of phytoplankton [e.g., diatoms, dinophytes, cyanobacteria, chlorophytes, haplotypes, cryptophytes (Everitt et al. 1990; Mackey et al. 1996; Wright et al. 1991]. The total chlorophyll *a* (Chl-*a*) content has been widely used as a proxy of phytoplankton biomass and the biomass of each phytoplankton group can also be quantified (Everitt et al. 1990; Yang et al. 2019). The analysis of pigments provides empirical data for calibrating remote sensing of functional types of phytoplankton in local and global oceans (Claustre et al. 2004; Sathyendranath et al. 2001). However, the ratio of cellular biomass in terms of carbon content to Chl-*a* content (C: Chl-*a*), an indicator of the physiological state of microalgae, varies greatly with environmental factors and among phytoplankton groups (Geider 1987; Sathyendranath et al. 2009). Furthermore, the taxonomic resolution of phytoplankton by chemotaxonomy is relatively low (class level, at best) (Eker-Develi et al. 2012).

Molecular approaches targeting nuclear 18S ribosomal RNA genes have been applied to characterize the diversity and community structure of unicellular eukaryotes at lower taxonomic ranks (e.g., family, genus, and even species levels) (Belevich and Milyutina 2022; Chen et al. 2019; Guo et al. 2016; Liu et al. 2021; Rii et al. 2018). With increasing sequencing depth and improvements in publicly available reference databases, rare eukaryotic taxa can be routinely sampled and classified (Tragin et al. 2018; Wang et al. 2022; Ye et al. 2022). Although metabarcoding of 18S rRNA genes covers both pigmented (phytoplankton) and non-pigmented eukaryotes (protozoa and fungi), the operational taxonomic units (OTUs) of eukaryotic phytoplankton taxa can be retrieved after classification to reassemble the eukaryotic phytoplankton communities (Kirkham et al. 2011; Trefault et al. 2021). For molecular quantification of phytoplankton eukaryotes, the 18S rRNA gene abundance of a taxon can be calculated by multiplying the total 18S rRNA gene copy number of the whole community (determined using quantitative real-time PCR assay, qPCR) with the sequence proportion of that taxon (Gong et al. 2020a; Zhu et al. 2005). There was a good correlation between the relative abundance of 18S rRNA genes and the proportion of pigment content of major phytoplankton groups in the Neuse River Estuary (Gong et al. 2020b); nevertheless, the relative abundance of 18S rRNA genes might not always reflect variations in pigment content, such as on the Western Antarctica Peninsula (Lin et al. 2019). It thus remains important to investigate the relationship between pigment content and rDNA abundance in different phytoplankton groups, which may have varying genome sizes across taxa (Lin 2011; Veldhuis et al. 1997). Furthermore, microalgal pigment content in phytoplankton species is well known to be cell size-dependent, and regulated by multiple environmental factors (e.g., light intensity, nutrient supply, and temperature) (Geider et al. 1986; Kana et al. 1997). Given that the relationship between 18S rRNA gene copy number and biovolume of a protistan cell follows a power law function, and the biovolume (a proxy of biomass) also varies with temperature (Fu and Gong 2017; Godhe et al. 2008), it is interesting to explore the relative importance of environmental conditions in modulating the rRNA gene abundance—pigment content relationship in communities of eukaryotic phytoplankton.

The 16S rRNA genes in chloroplast genomes of eukaryotes and Cyanobacteria can be targeted and amplified using plastid-specific PCR primers (Decelle et al. 2015). In contrast to the high levels and large variability of copy numbers of 18S rRNA genes in eukaryotic genomes (Gong et al. 2013; Salmaso et al. 2020), the copy numbers of plastid 16S rRNA genes per cell are relatively low and constant (Bennke et al. 2018; Shi et al. 2011), which raises the question of how different the diversity and community structure are when determined by these two molecular markers. Recently, comparative studies targeting both plastid 16S and 18S rRNA genes have found similar temporal patterns of phytoplankton community structure (Needham and Fuhrman 2016), and good correlations between plastid 16S and 18S rRNA gene abundances of cryptophytes and three diatom species (Lin et al. 2019), and between 16S- and 18S-based ratios of relative abundance of many shared phytoplankton classes (Yeh and Fuhrman 2022). However, the quantitative relationships between plastid rRNA gene abundances and pigment contents in specific phytoplankton groups, whether the 16S- and 18S-based alpha diversity estimators of eukaryotic phytoplankton are consistent in spatiotemporal patterns, and which taxa are strongly biased in the 16S- or 18S-based phytoplankton communities, against those characterized using pigments, still need to be investigated.

**Fig. 1:**
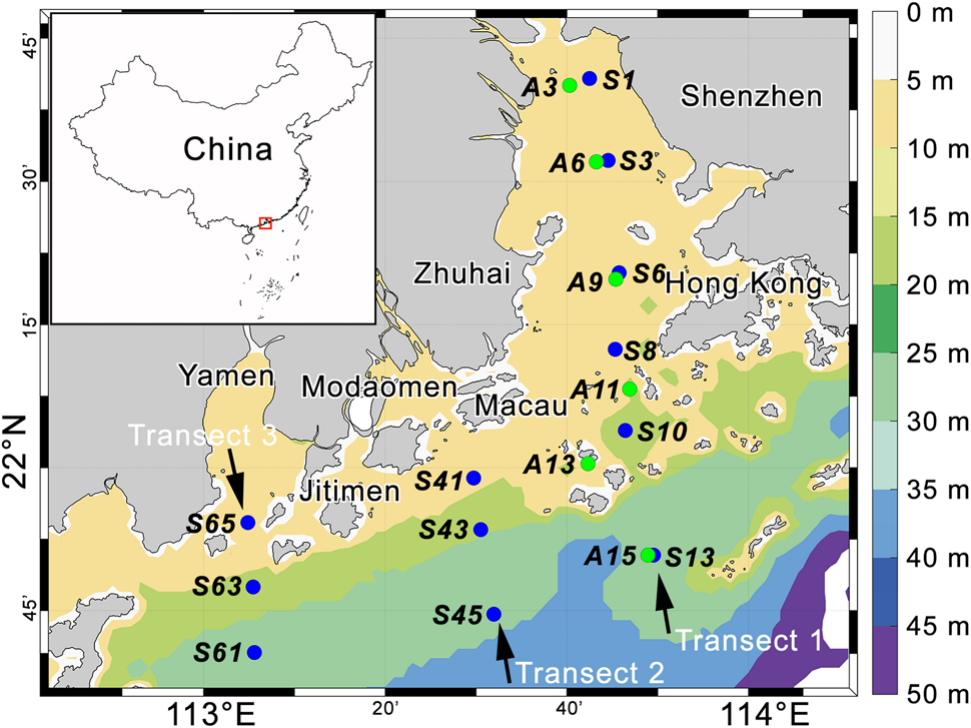
A map showing sampling sites in Transects 1–3 in the Pearl River Estuary, South China Sea. Twelve sites (depicted in blue) and 6 sites (in green) were visited in July and November, respectively. The color bar shows the water depths

The Pearl River Estuary (PRE) is a tropical estuary off the South China Sea, where phytoplankton blooms in the middle part of the estuary during the dry season (Jia et al. 2019; Lu and Gan 2015; Niu et al. 2020; Qiu et al. 2019). Phytoplankton diversity, abundance, and community structure have been studied based on either morphological identification (Chen et al. 2019; Jiang et al. 2015), 18S rRNA gene sequencing (e.g., Wu and Liu 2018), or pigment analysis (Chai et al. 2016). In this study, we investigate the spatial and seasonal patterns of eukaryotic phytoplankton in the PRE using a combination of three methodologies: pigment analysis and molecular approaches targeting both nuclear and plastid rRNA genes. The main aims were: (1) to assess the quantitative linkages between nuclear and plastid rRNA gene abundances and pigment contents of eukaryotic phytoplankton; (2) to quantify the relative importance of environmental factors in explaining the variation in the ratio of 18S rRNA gene copy number (log-transformed) to the content of Chl-*a* (a proxy of C: Chl-*a*); and (3) to explore potential advantages and limitations of the two molecular approaches in covering alpha and beta diversity of phytoplankton groups (Fig. 1).

## Results

### Hydrological parameters

**Fig. 2:**
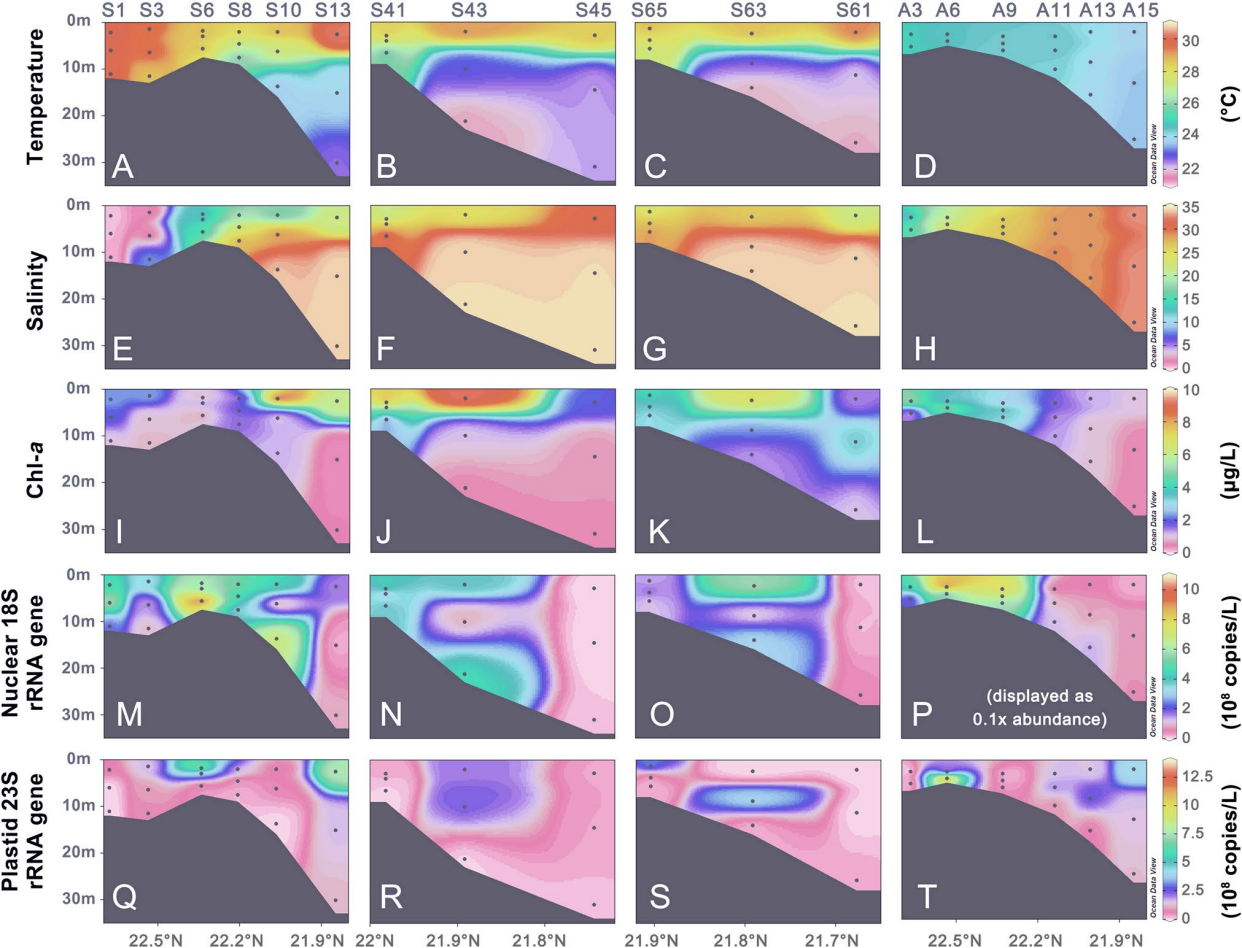
Vertical profiles of temperature (**A–D**), salinity (**E–H**), total chlorophyll *a* (Chl-*a*, **I–L**), nuclear 18S rRNA gene (**M–P**), and plastid 23S rRNA gene abundances (**Q–T**) of eukaryotic phytoplankton along three summertime transects (including sites S1–S13, S41**–**S45, and S65**–**S61) and a wintertime transect (including sites A3**–**A15) in the Pearl River Estuary

**Fig. 3:**
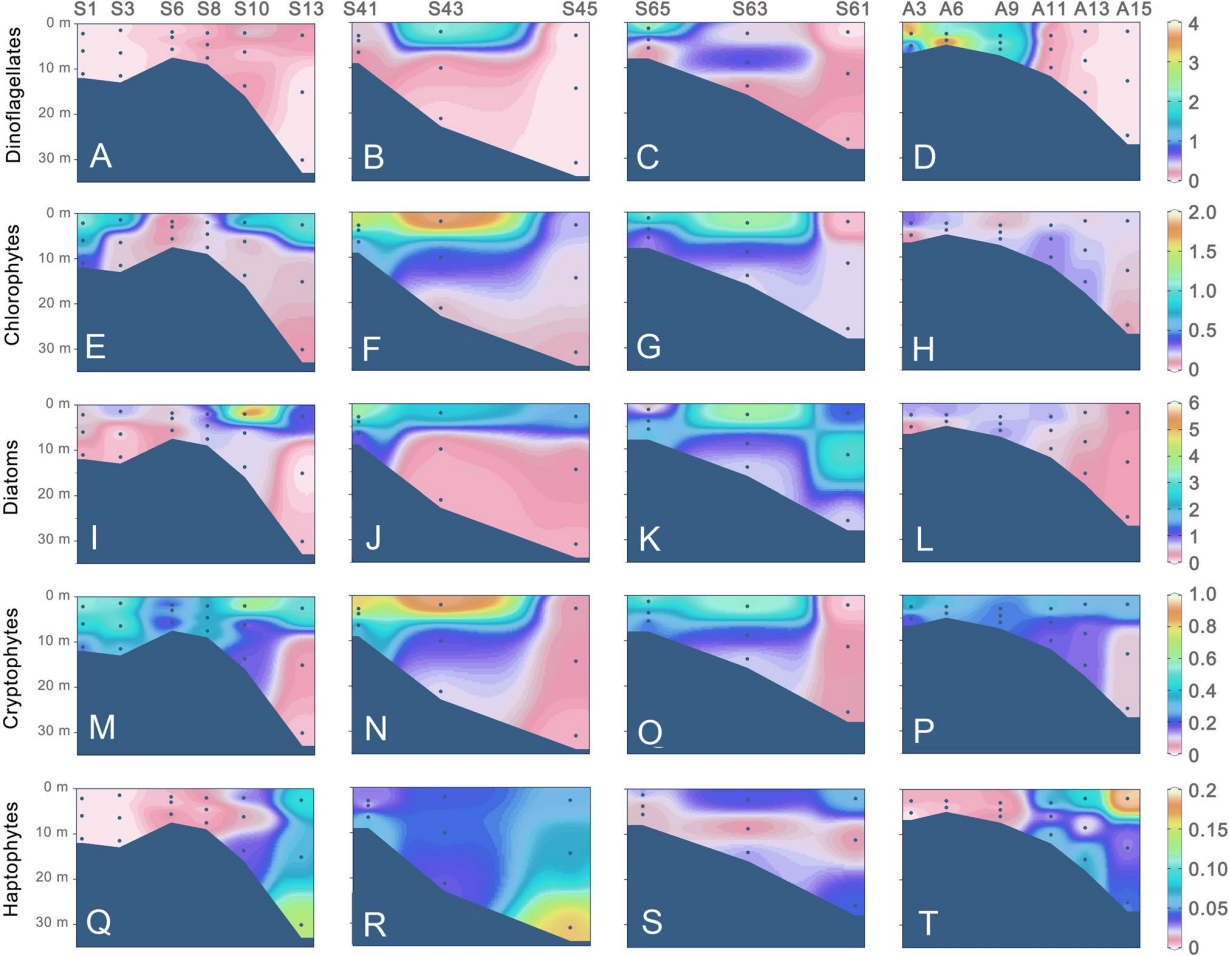
Vertical distribution of chlorophyll *a* content (μg/L) contributed by the five major phytoplankton groups, pigmented dinoflagellates (**A–D**), chlorophytes (**E–H**), diatoms (**I–L**), cryptophytes (**M–P**), and haptophytes (**Q–T**)

The profiles of temperature, salinity and Chl-*a* in the PRE showed significant spatial and seasonal variability (Fig. 2A–L). The water column was distinctly stratified in July, with high temperatures (28–30.2 °C) in the 10-m surface layer and lower temperatures (22–24 °C) below a depth of 15 m (Fig. 2A–C). The thermal stratification disappeared in November, with uniform temperature ranging from 23.6 to 25.0 °C (Fig. 2D). A saltwater wedge was distinct in all transects and both seasons (Fig. 2E–H). Along Transect 1, the freshwater tougue extended further seawards in July than in November. The highest total Chl-*a* concentration (determined by HPLC) in surface waters occurred along an arc linking the sites S10 (9.13 μg/L), S43 (9.18 μg/L), and S63 (6.93 μg/L; Fig. 2I–K) in July, which corresponded to a diatom bloom (Fig. 3I–K). However, the high levels of surface Chl-*a* (up to 3.2 ~ 5.4 μg/L) occurred at the inner estuary in November (Fig. 2L), indicating phytoplankton blooms in that area.

The concentration of dissolved oxygen (DO) ranged from 0.55 to 6.11 mg/L in the summer with much lower values (< 3 mg/L) in the bottom waters (depth of 10–20 m) at sites S8, S10, S41, S43, and S63 (Supplementary Fig. S1). The bottom hypoxic zones usually co-occurred when there were phytoplankton blooms in the surface waters. In general, the concentrations of NO_3_^−^ (0.21–138.64 μmol/L), NO_2_^−^ (0.01–16.61 μmol/L), soluble reactive phosphorus (SRP, 0.02–1.11 μmol/L), and dissolved silicate (DSi, 3.95–107.96 μmol/L) varied markedly in space and between seasons but generally decreased from freshwater to more saline sites. However, NH_4_^+^ concentration (0.97–11.86 μmol/L) was generally higher at the more saline sites and in the deeper layers (Supplementary Fig. S1).

### Nuclear 18S and plastid 23S rRNA gene abundances

The distribution of both nuclear 18S and plastid 16S rRNA gene copy numbers of eukaryotic phytoplankton was uncoupled with the concentration of Chl-*a* along the PRE transects (Fig. 2M–T). Unlike Chl-*a* that peaked at the surface, the 18S rRNA genes were most abundant at the bottom in Transects 1 and 2 (up to 7.5 × 10^8^ and 3.2 × 10^8^ copies/L). Nevertheless, for the samples collected at Transect 3 in July and Transect 1 in November, the higher abundances of 18S rRNA genes (4.2 × 10^9^ copies/L) were coincident with higher levels of Chl-*a* (Fig. 2M–P). Unlike Chl-*a* and 18S rRNA genes, the plastid 23S rRNA gene abundance was not that variable in space (0 ~ 6.52 × 10^8^ copies/L), with peaks in a few surface samples and shallow sites in Transect 1 in both seasons (Fig. 2Q–T).

### Distribution of major phytoplankton groups based on pigment content

The Chl-*a* contents of dinophytes, chlorophytes, diatoms, cryptophytes, and haptophytes exhibited high variability across the transects and the two seasons (Fig. 3A–T). Spatially, all groups were abundant in surface waters (depth < 10 m), except for Haptophyta, which had the highest biomass (0.14 to 0.16 µg Chl-*a*/L) in the bottom waters at two marine sites (S13 and S45 with depths about 30 m; Fig. 3Q, R). During the summer cruise, the eukaryotic phytoplankton pigment was dominated by diatoms (0.03 – 5.96 µg Chl-*a*/L), followed by dinophytes (0–1.75 µg Chl-*a*/L) and chlorophytes (0.03–1.80 µg Chl-*a*/L); cryptophytes (0.02–0.94 µg Chl-*a*/L), and haptophytes (0–0.16 µg Chl-*a*/L) were minor. In November, dinophytes bloomed at the less saline sites of Transect 1, contributing up to 4.3 µg Chl-*a*/L (Fig. 3D), and other groups contributed only small fractions to the surface Chl-*a* (Fig. 3H, L, P, T).

### Environmental factors influencing the relationships among pigments, nuclear and plastid rRNA abundances

**Fig. 4:**
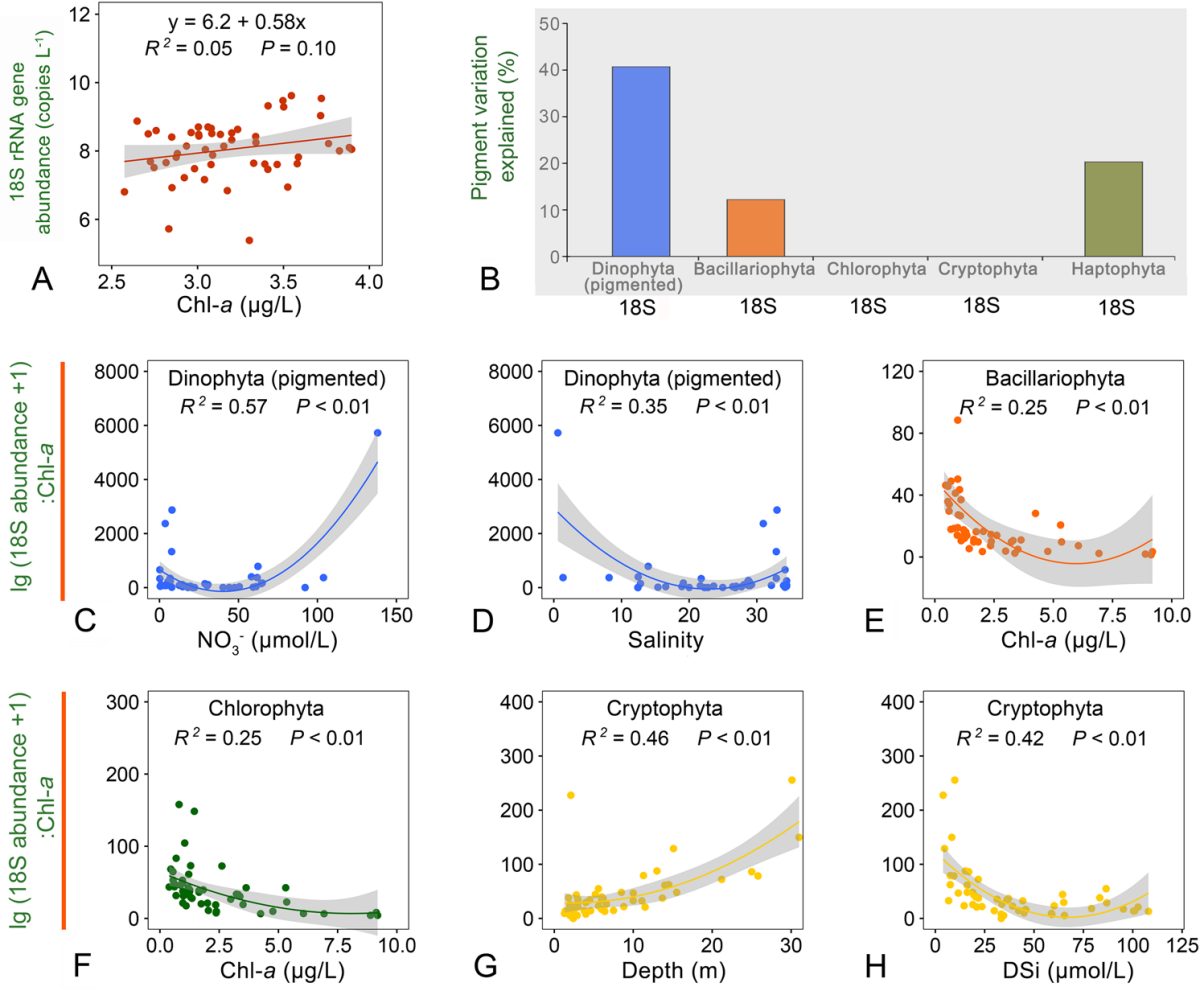
Regression analysis showing significant relationships between ratio of 18S rRNA gene abundance (log(*x* + 1) transformed) to Chl-*a* and other environmental factors. Chl-*a*, chlorophyll *a*; DSi, dissolved silicate

There was no significant correlation between total Chl-*a* and 18S rRNA gene abundance of eukaryotic phytoplankton (*P* > 0.05; Fig. 4A). The 18S abundance explained 41% of the variability of the pigmented dinophyte Chl-*a* content (*P* < 0.05). For other groups, the explanatory power reduced greatly, being 20% and 12% for haptophytes and diatoms, respectively (*P* < 0.05), and none for chlorophytes and cryptophytes (*P* > 0.05; Fig. 4B). The correlations between 18S and plastid 23S rRNA gene abundances and pigment contents were usually weak or insignificant for diatoms, chlorophytes, cryptophytes and haptophytes, but stronger for dinoflagellates (0.17 < *R*^2^ < 0.42, *P* < 0.05; Supplementary Fig. S2).

The ratios of 18S rRNA gene abundance (log(*x* + 1) transformed) to Chl-*a* content of the four microalgal groups varied widely across season and space, ranging from 2 to 5726 (on average 398) in the pigmented dinophytes, and from 1 to 88 in diatoms (average 22), chlorophytes (average 40), and cryptophytes (average 45) (Supplementary Fig. S3). Regression analysis indicated that the ratio for pigmented dinophytes was significantly related to NO_3_^−^ (*R*^2^ = 0.57; *P* < 0.01) and salinity (*R*^2^ = 0.57; *P* < 0.01); Fig. 4C, D). The 18S to Chl-*a* ratio in both diatoms and chlorophytes tended to decrease with increasing total phytoplankton biomass (*R*^2^ = 0.25; *P* < 0.01; Fig. 4E, F). In contrast, the cryptophytes had a higher level of 18S rRNA gene abundance per μg Chl-*a* in a deeper layer (*R*^2^ = 0.46; *P* < 0.01), or in waters with a lower concentration of DSi (*R*^2^ = 0.42; *P* < 0.01; Fig. 4G, H).

### Diversity and taxonomic composition based on 18S, 16S, and pigment analysis

**Fig. 5:**
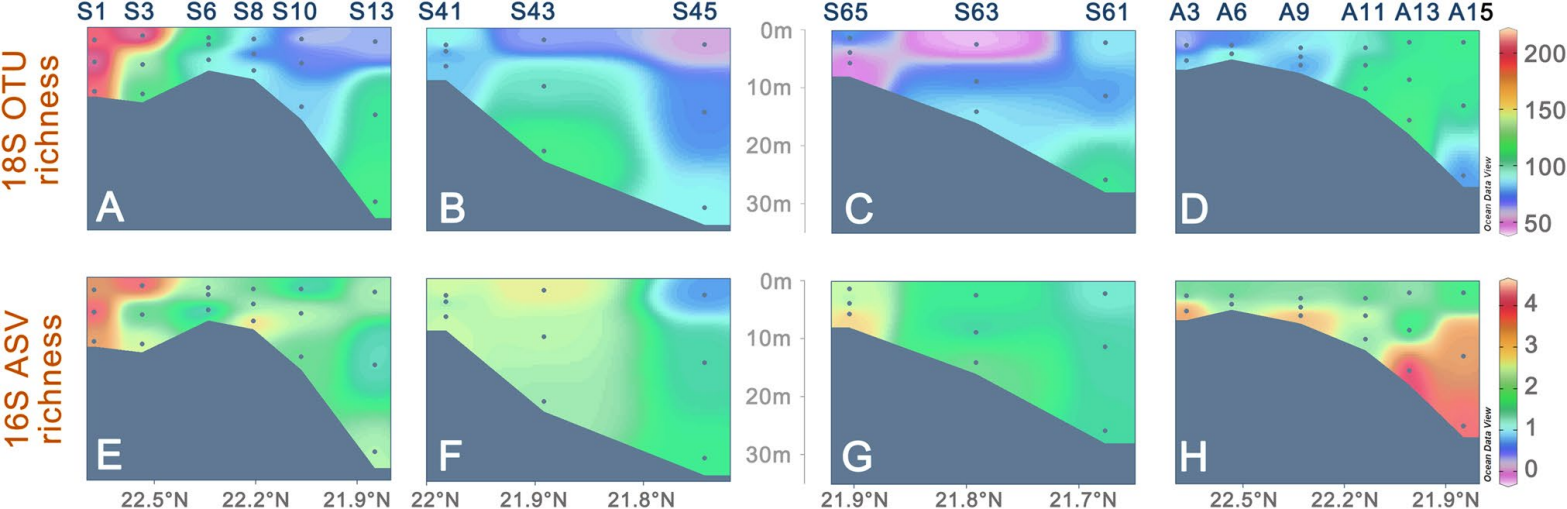
Spatial and seasonal variations in 18S-based OTU richness (**A–D**) and 16S-based ASV richness (**E–H**) of eukaryotic phytoplankton along the three transects. ASV, amplicon sequence variant; OTU, operational taxonomic unit

The raw sequencing data (4,635,358 reads of 18S rRNA genes and 4,755,849 reads of 16S rRNA genes) were processed and analyzed using QIIME2. A total of 356,968 and 550,538 reads were retained for nuclear 18S and plastid 16S rRNA genes after quality control, respectively. A total of 712 OTUs and 5531 amplicon sequence variants (ASVs) were detected for 18S and 16S rRNA genes, respectively. By rarefying at 3789 reads per sample for 18S and 3000 reads per sample for 16S, the richness of both molecular markers in surface waters was mapped, showing a decrease towards the sea in July (Fig. 5A–C, E–G), but the opposite trend in November (Fig. 5D, H).

**Fig. 6:**
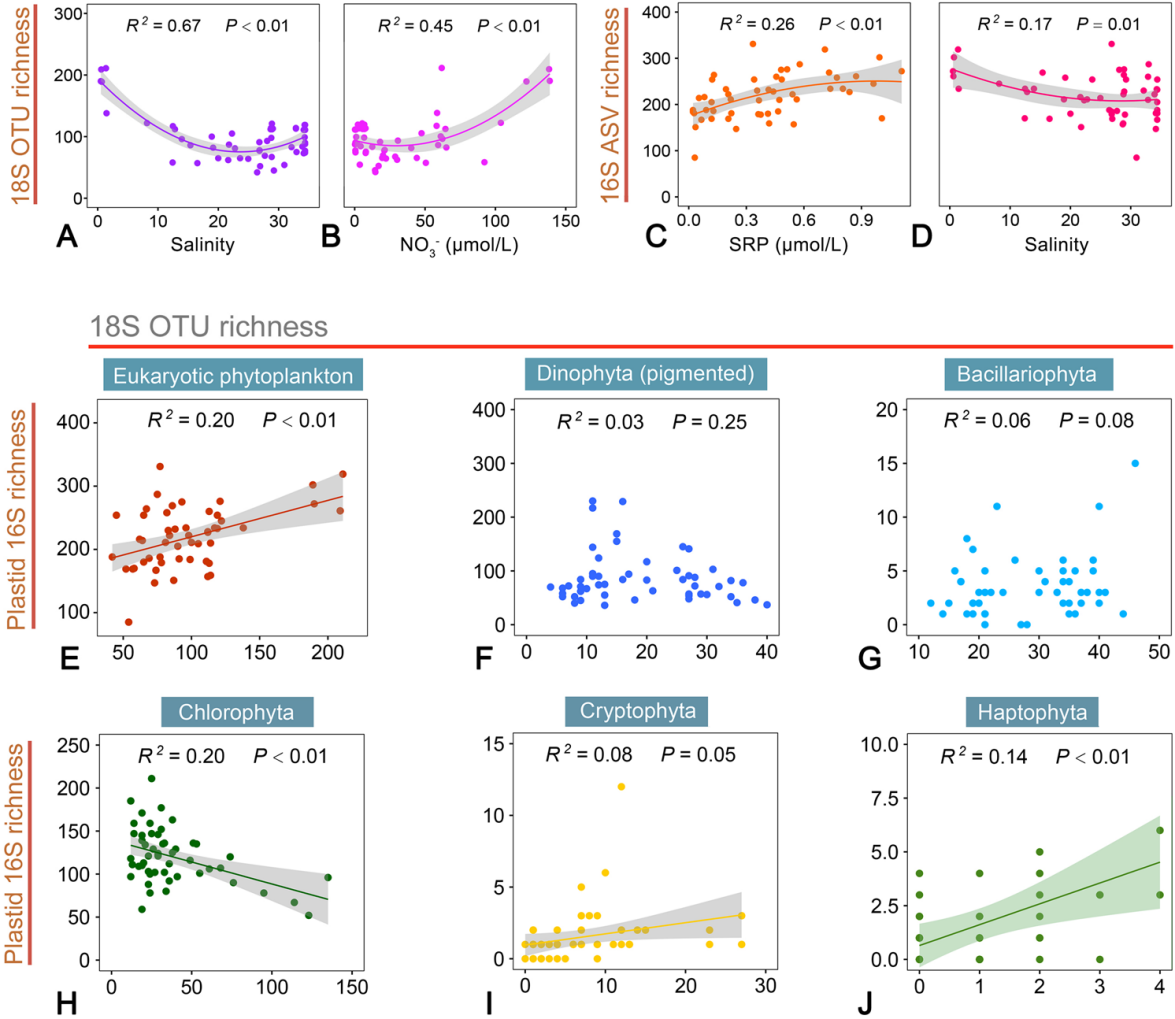
The relationships between 18S-based OTU richness and the identified environmental factors that were most significant in the regression analyses (**A–D**). Also note that the weak correlations between 18S-based OTU and 16S-based ASV richness of the whole eukaryotic phytoplankton communities (**E**), and of individual phytoplankton groups (**F–J**). ASV, amplicon sequence variant; OTU, operational taxonomic unit; SRP, soluble reactive phosphorus

Regression analyses showed that the importance of environmental factors in driving the alpha diversity of eukaryotic phytoplankton was different when different molecular markers were considered (Fig. 6A–D, Supplementary Fig. S4). Salinity (*R*^2^ = 0.67, *P* < 0.001) and NO_3_^−^ (*R*^2^ = 0.45, *P* < 0.001) were the most significant environmental factors in explaining the variation in 18S-based OTU richness and Chao1 index (Fig. 6A, B, Supplementary Fig. S4B), whereas SRP (*R*^2^ = 0.26, *P* < 0.001) and salinity (*R*^2^ = 0.17, *P* < 0.001) were most significant for 16S-based ASV richness (Fig. 6C, D).

Linear regression analysis showed that 18S OTU richness of eukaryotic phytoplankton was positively related to plastid 16S ASV richness (slope = 0.576), and explained 20% of the variation across all samples examined (*P* < 0.01; Fig. 6E). However, for an individual group of phytoplankton, the regressions were either insignificant (diatoms and dinophytes, *P* > 0.05; Fig. 6F, G) or much weaker (cryptophytes and haplotypes, Fig. 6I, J). There was even a negative relationship between the richness of these two markers in chlorophytes (Fig. 6H). Correlations between other 16S- and 18S-based alpha diversity indices of eukaryotic phytoplankton or an individual group were weak or insignificant too (Supplementary Figs. S5, S6).

**Fig. 7:**
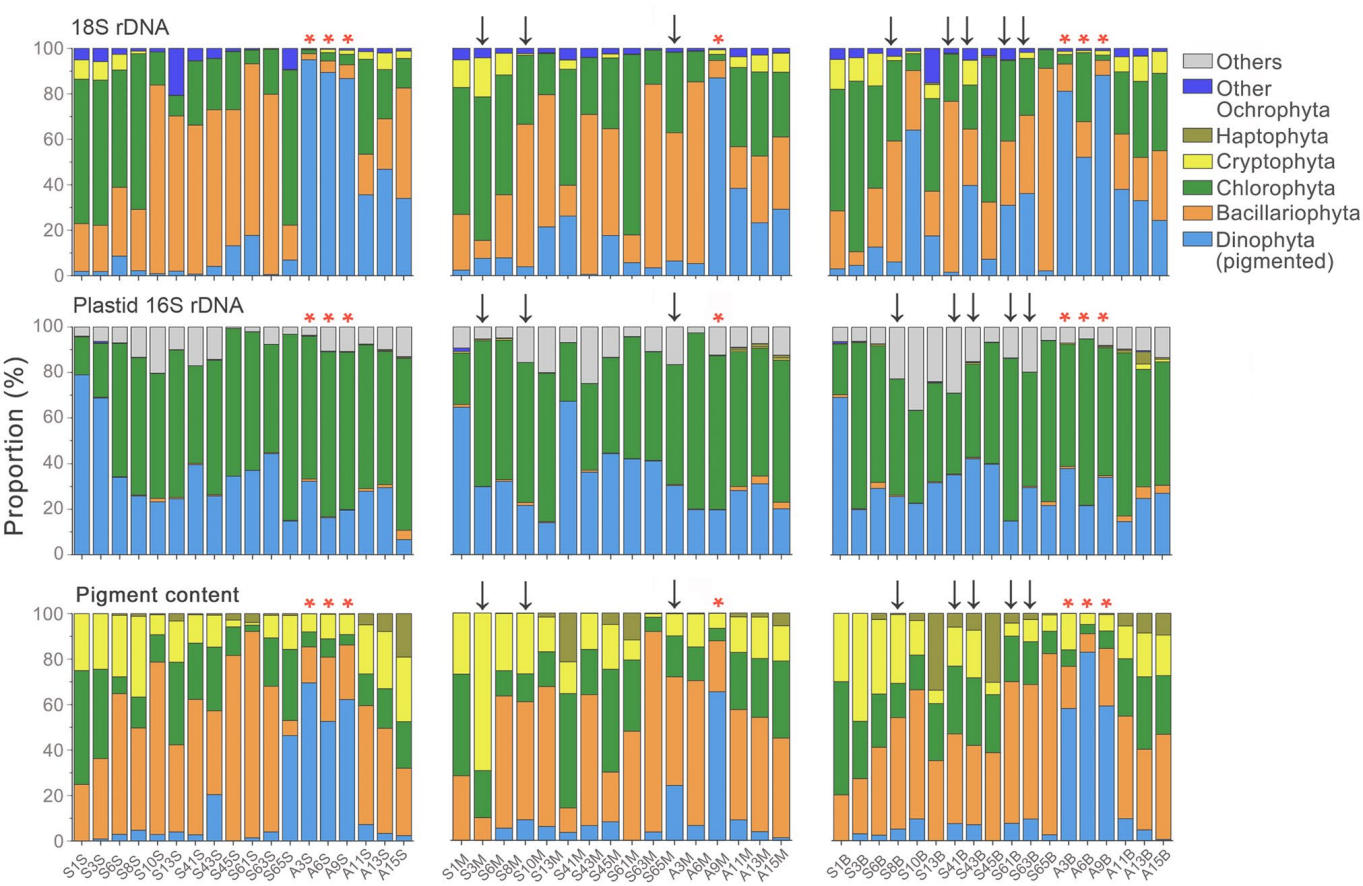
Community composition of eukaryotic phytoplankton in surface (left panel), middle layer (middle panel) and bottom waters (right panel) in the Pearl River Estuary, as revealed by high throughput sequencing of nuclear 18S rRNA genes (upper row), plastid 16S rRNA genes (middle row), and pigment analysis (bottom row). Arrows and asterisks indicate the samples where hypoxia and blooms of pigmented dinophytes occurred

As far as the five major microalgal groups were concerned, the community composition of eukaryotic phytoplankton characterized using the plastid 16S rRNA gene was quite different from those documented by metabarcoding 18S rRNA genes and using pigment analysis, whereas the latter two methods appeared to yield highly concordant results (Fig. 7). Most plastid 16S rRNA gene reads were affiliated with Chlorophyta (on average 55%) and pigmented Dinophyta (32%). Bacillariophyta (< 5%) and Cryptophyta (< 1.2%), which had much lower proportions in 16S-based communities, however, appeared to be much more abundant in both 18S (average 36% and 3%) and pigment (average 43% and 18%) datasets. The haptophyte pigment was frequently abundant (0 – 34%) in the HPLC samples, but this group was rarely identified in the plastid 16S (< 1.3%), and hardly detected (< 0.7%) in the 18S dataset. Other Ochrophyta (i.e., the ochrophytes not including diatoms) frequently occurred with low to moderate proportions of reads (0.2% – 21%) in the 18S dataset, however, was detected in only a few samples by 16S sequencing, and was never identified by pigment analysis (Fig. 7). Blooms of dinophytes in seven samples (Chl-*a* percentages > 50%, and total Chl-*a* > 1 µg/L) were well reflected by their high sequence proportions in the 18S dataset (asterisks in Fig. 7). However, these signals of dinophyte blooms were not distinguishable in the 16S dataset. In hypoxic waters, pigment-based biomass of chlorophytes appeared to be lower than those in normoxic samples, which, however, was not pronounced in the 18S and 16S datasets (arrows in Fig. 7).

The community composition of eukaryotic phytoplankton resolved at the order level showed that some orders were identified only in one of the 18S and 16S datasets (Supplementary Fig. S7). For example, orders Gymnodiniales and Suessiale, Prorocentrales, and Gonyaulacales were not resolved in the 16S (Supplementary Fig. S7A–C). Two diatom species *Cyclotella* sp. (Order Thalassiosirales) and *Guinardia delicatula* (Order Rhizosoleniales) appeared to be dominant in the 18S dataset but were not identified from the 16S (Supplementary Fig. S7D–F). The most dominant taxa within cryptophytes were assigned to two different orders in 18S (Order Cryptomonoadeles) and 16S datasets (Order Pyrenomonadales) (Supplementary Fig. S7J–L).

**Fig. 8:**
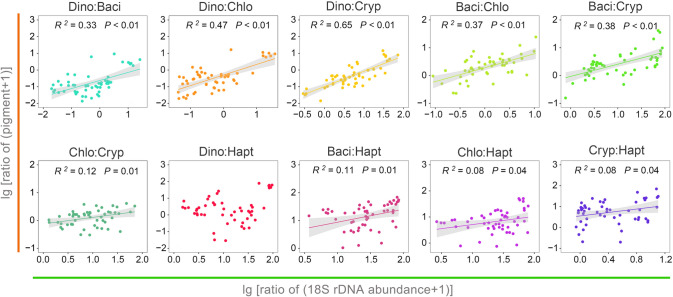
Scatter plots showing linear regressions between ratio of 18S rRNA gene abundance of two major microalgal groups and ratio of pigment contents of that microalgal pair. Dino, pigmented dinophytes; Baci, Bacillariophyta; Chlo, Chlorophyta; Cryp, Cryptophyta; Hapt, Haptophyta

Regression analysis showed that the ratio of 18S rRNA gene abundances between two of the four microalgal groups generally related well to their ratio of Chl-*a* contents (0.3 < *R*^2^ < 0.66, *P* < 0.05; Fig. 8). However, the correlations between chlorophytes and cryptophytes (*R*^2^ = 0.12, *P* < 0.01), and between haptophytes versus all other groups were weak (0.05 < *R*^2^ < 0.12; Fig. 8). There were no significant relationships between the ratio of 18S and plastid 16S rRNA gene abundance of any two of the five major microalgal groups (Supplementary Fig. S8A). The relations between the ratio of plastid 16S and the ratio of pigment content were weak or not significant (Supplementary Fig. S8B).

**Fig. 9:**
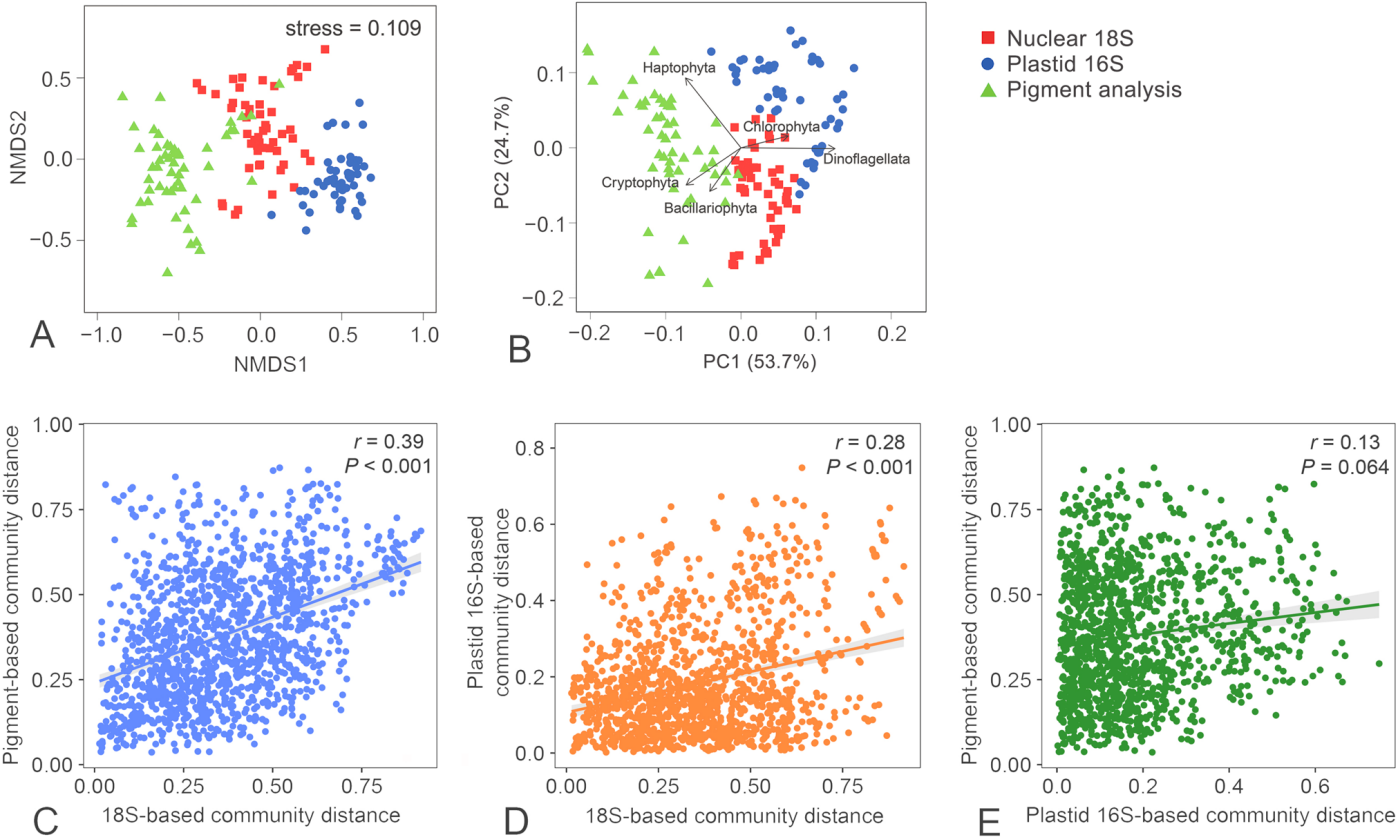
Variations in phytoplankton community composition as characterized using nuclear 18S, plastid 16S, and pigment-based methods. A non-metric multidimensional scaling (NMDS) plot showing the variations in phytoplankton community structure resolved at the phylum level (**A**). A plot of principal component analysis showing that Haptophyta was underestimated in the 18S dataset, whereas diatoms and cryptophytes underestimated in the plastid 16S dataset (**B**). Scatter plots showing Pearson correlations between phytoplankton community distances based on 18S, plastid 16S, and pigments (**C–E**)

The community structure of eukaryotic phytoplankton based on the five major microalgal groups revealed using plastid 16S rRNA genes were much more different from those assessed by pigment analysis than by metabarcoding 18S rRNA genes (Fig. 9A). Compared with the community structure assessed by pigment contents, chlorophytes, and pigmented dinoflagellates tended to be overestimated in both 18S and 16S based communities, whereas cryptophytes might be underestimated in 18S-based communities. The metabarcoding of 18S rRNA genes, however, suffered from bias against haptophytes, which might be underestimated in 16S-based phytoplankton community as well (Fig. 9B). Pairwise correlations of community distance obtained from different methods also supported that 18S rRNA gene could reflect more variability in pigment-based community structure of eukaryotic phytoplankton in the PRE (*r* = 0.39, *P* < 0.001) than plastid 16S rRNA genes (*r* = 0.13, *P* = 0.064) (Fig. 9C–E).

### Environmental factors affecting the community structure of eukaryotic phytoplankton

**Fig. 10:**
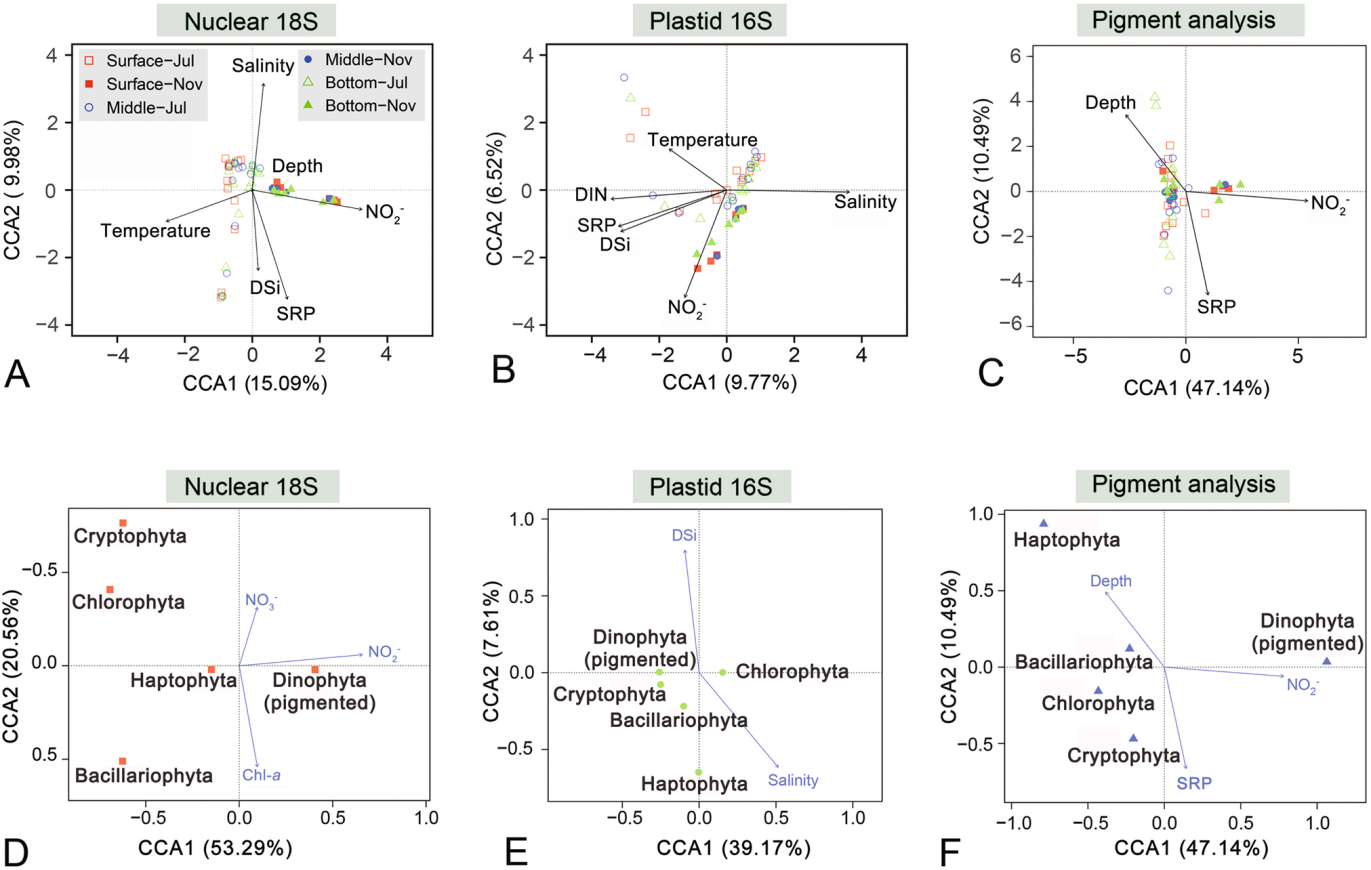
Plots of canonical correspondence analysis (CCA) showing the environmental factors that significantly varied with the community structure of eukaryotic phytoplankton (**A–C**), and with relative abundances of major phytoplankton groups. The community structure was based on 18S (**A** and **D**), 16S rRNA genes (**B** and **E**), and pigments (**C** and **F**), and resolved at the OTU (**A**) and ASV (**B**) levels, and at major group levels (**C–E**). Note that different sets of environmental factors were selected for the phytoplankton communities characterized using these methods, nevertheless, more similar sets of environmental variables were selected in those characterized by 18S sequencing and pigment analysis. The water depths (surface, middle, and bottom layers) and months (July and November) of the collected samples are annotated in (**A–C**). Chl-*a*, chlorophyll *a*; DIN, dissolved inorganic nitrogen; DSi, dissolved silicate; SRP, soluble reactive phosphorus

The CCA plots showed that the measured environmental factors could explain a small portion of variations in both 18S-OTU and plastid 16S-ASV-based community structure of eukaryotic phytoplankton, for which NO_2_^−^, temperature, salinity, and nutrients were the most significant driving factors (Fig. 10A–B). However, NO_2_^−^, depth and SRP were found to be significant factors driving the changes in the eukaryotic phytoplankton community based on the pigment proportions of the five major groups (Fig. 10C). The higher concentration of NO_2_^−^, the higher proportion of 18S and pigment-based biomass of dinophytes (Fig. 10D, F). Relative quantities of the other four phytoplankton groups were mainly influenced by Chl-*a* and NO_3_^−^ in 18S-based communities (Fig. 10D), and by SRP and depth in pigment-based communities (Fig. 10F). In contrast, totally different environmental variables (salinity and DSi) were selected as the major driving factors for phytoplankton community in the application of plastid 16S approach (Fig. 10E).

## Discussion

The spatial and seasonal variations in the community composition of eukaryotic phytoplankton in the tropical PRE provided a good experimental field to examine the consistency and discrepancy of different approaches in characterizing quantity and diversity. Basically, the 18S and pigment-based data of 2020 were consistent with existing surveys of phytoplankton in the PRE using morphological observations. For example, all the studies of Jia et al. (2019), Xu et al. (2022), and Zhong et al. (2020a) demonstrated that diatoms were the dominant phytoplankton group in July, followed by chlorophytes. Dong et al. (2020) observed a bloom of the dinoflagellate *Cochlodinium geminatum* in November 2018. Similarly, a dinoflagellate bloom (likely *Polykrikos geminatus*) was observed in November 2020, which led to significant high 18S sequence proportions of dinoflagellates (> 50%) at the stations A3, A6 and A9 (Fig. 7).

It was found that the 18S-based community structure of eukaryotic phytoplankton was highly concordant with that based on pigment content. This judgment is based on at least the following three facts: (1) the rRNA gene abundance ratio between any two groups of dinophytes, chlorophytes, diatoms, and cryptophytes generally well reflected the ratio of their pigment contents (Fig. 8); (2) the dinophyte blooms identified in the pigment analysis were also well captured in the 18S dataset (Fig. 7); and (3) 18S- and pigment-based community distances were well correlated with each other (Fig. 9C). This result is consistent with Gong et al. (2020b), who demonstrated similar relationships between 18S rRNA gene abundance and pigment content in a study of the community structure of eukaryotic phytoplankton in a temperate estuary of USA. Lin et al. (2019) even found that the absolute abundance of 18S rRNA genes was an even better correlate of pigment contents of cryptophytes, diatoms, and *Phaeocystis* in a coastal region of the Antarctic. Since cellular Chl-*a* content has long been known to be a good indicator of microalgal biomass, these results indicate that the 18S rRNA gene-based community structure approximates a biomass-based community structure of eukaryotic plankton. This notion is supported by the finding of power-law relationships between cellular 18S rRNA gene copy number and cell biovolume in dinoflagellates (Godhe et al. 2008), and several heterotrophic protistan species (Fu and Gong 2017; Zou et al. 2021).

It was found that plastid 16S ASV richness was positively related to 18S OTU richness across all samples, suggesting co-evolution between plastid and nuclear genes in eukaryotic phytoplankton, as previously demonstrated for plants (Forsythe et al. 2020). Nevertheless, despite a looser definition of “OTU” (i.e., ASV) being applied for 16S than 18S, the 16S richness varied to a lesser extent (with a slope of regression < 1) than the 18S OTU richness, an explanation for which is that plastid 16S rRNA genes are generally more conservative than nuclear 18S rRNA genes in reflecting phytoplankton diversity. Similar results on lower evolutionary rates of plastid genes relative to nuclear ones have been noted in plants (Drouin et al. 2008; Wolfe et al. 1987). Therefore, the use of plastid markers for assessing phytoplankton diversity may be compensatory to the approach of nuclear 18S rRNA genes of eukaryotic phytoplankton (particularly for the lineages which were commonly detected from both 16S and 18S datasets), which frequently leads to an overestimation of species diversity due to intragenomic polymorphisms (Zou et al. 2021).

It has long been known that the ratio cellular C: Chl-*a* is a sensitive indicator of the physiological state of phytoplankton, usually ranging from 10 to 130, and tending to be higher in larger cell size, at higher levels of irradiance, and nitrate availability and growth rate, and at lower temperatures (Geider et al. 1986; Taylor et al. 1997). The ratio also differs between microalgal groups, with the value increasing in the following order: chlorophytes < diatoms < dinophytes (Geider et al. 1986). The 18S copy number per unit Chl-*a* was also found to be highest in dinophytes. Moreover, higher levels of 18S: Chl-*a* of dinophytes occurred in the waters with higher nitrate, and higher 18S: Chl-*a* ratios of diatoms and chlorophytes were detected in the samples with lower total Chl-*a* in bottom waters (Fig. 4C–F), which was consistent with the negative relationships between C: Chl-*a* and total Chl-*a* in the North Atlantic (Taylor et al. 1997) and with the environmental effects on C: Chl-*a* discussed above. The reason for this consistency may be that the cellular 18S rRNA gene copy number of protists scales with cell biovolume (volume-based biomass), as shown for protist species (Fu and Gong 2017; Godhe et al. 2008; Zou et al. 2021). However, what was not expected was that the ratio of cryptophyte 18S: Chl-*a* was higher at the bottom waters, where light availability was low, which is contradictory to the usual increase in pigment content by photoadaptation in low light (Geider et al. 1986; Kana et al. 1997). A possible explanation for this observation is that cryptophytes have accessory pigments such as phycoerythrin and phycocyanin, which enable high photo-absorption and growth rates in red- and blue-light dominated environments, such as at depth in estuaries (Heidenreich and Richardson 2020). Furthermore, the mixotrophic cryptophytes can adapt by shifting to a heterotrophic lifestyle as bacterial grazers (Hansen et al. 2019), when the irradiance became limiting for active photosynthesis. In short, this study suggests that the ratio 18S: Chl-*a* could be a potential alternative to C: Chl-*a* in correcting and modeling pigment-based biomass of phytoplankton.

The proportion of haptophytes was less than 0.7% in the 18S dataset, although this could be underestimated, considering the pigment-based biomass of haptophytes in PRE was up to 0.2 μg Chl-*a*/L, and 34% of the eukaryotic phytoplankton community in the present study and in a previous investigation (Chai et al. 2016). This underestimation of haptophytes in the 18S dataset could be due to the eukaryote-universal 18S rRNA gene primers being strongly biased against Prymnesiales (Haptophyta) (Liu et al. 2009; Yeh et al. 2021), and their higher GC content (57%) in 18S than many other groups, which undermines the efficiency of PCR amplification (Liu et al. 2009). This bias could also explain the observations that the 18S ratio of haptophytes and other microalgal groups poorly reflects their pigment-based biomass proportions (Fig. 8). Similarly, the cryptophyte biomass (average 18%) might also be underestimated in the 18S dataset (average 3%). Since the 18S ratio of cryptophytes to other groups was well correlated with their pigment ratio (Fig. 8), indicating other cellular characteristics (e.g., having fewer rRNA gene copies per cell than other microalgae) may underlie the disproportional rRNA gene of this group. Given the importance of haptophyte and cryptophyte biomass in the phytoplankton of many PRE samples, these two groups could be investigated using group-specific primers targeting 18S rRNA genes; cellular rRNA gene copies have yet to be quantified for a better understanding of their diversity and quantity in coastal systems.

The plastid 16S-based community structure of eukaryotic phytoplankton was much different from those of both the pigment- and 18S-based structures. It was also demonstrated that 515F and 926R have good coverage for the plastid 16S (Mcnichol et al. 2021). Similar to a previous study that found contrasting relative abundances of diatoms and cryptophytes in 18S and 16S datasets across some Antarctic samples (Hamilton et al. 2021), the proportions of diatoms, cryptophytes, haptophytes in the 16S databases were also found to be much lower than those in the 18S-based datasets. There are many 18S rRNA gene copies (thousands to hundreds of thousands) in a microalgal cell, whereas the plastid 16S rRNA gene copy numbers have been reported to be much lower and less variable, often one to few dozen copies per genome in some phytoplankton species (Bennke et al. 2018; Needham and Fuhrman 2016). This suggests that 16S-datasets likely approximate cell abundance-based structures rather than a biomass-oriented organization of eukaryotic communities, to which pigment and 18S datasets are more relevant. Nevertheless, the copy number of plastid 16S of more phytoplankton species, and the relationship between cell abundance and total plastid 16S copy number has yet to be further experimentally explored. Furthermore, there were discrepancies in identifying lower-ranked taxa using these two markers in this study. Only a few phytoplankton genera were commonly detected (Trefault et al. 2021). The differences in recovering lower-ranked taxa could contribute to not only their proportional differences in the communities but also the diversity estimators using 18S and 16S rRNA gene sequencing. In addition, it should be noted that, although the pigmented dinophyte 16S appeared to be abundant, underestimation of their abundance is still possible, since the plastid rRNA genes of many dinoflagellates are difficult to amplify and there are not many sequences curated in the PhytoREF database (Decelle et al. 2015).

## Concluding remarks

The molecular and pigment data collected from a tropical estuary were analyzed to explore whether similar variational patterns in diversity, quantity, and community structure of eukaryotic phytoplankton could be obtained using different methodologies. In general, it was found that there were insignificant or poor correlations among 18S rRNA, plastid 23S rRNA gene abundance, and Chl-*a* content, and between richness of 18S OTUs and plastid 16S ASVs of eukaryotic phytoplankton. The 18S- and the pigment-based community structure were more similar to each other than to the 16S-based structure. Not surprisingly, these inconsistencies resulted in different sets of major environmental drivers being identified for the datasets obtained using 18S, 16S, and pigment approaches. The discrepancies between the two molecular approaches might be caused by primer bias, different genome sizes and gene copy numbers among phytoplankton groups, and insufficient reference sequences with high taxon coverage in the database. The Chl-*a*-proxied biomass of phytoplankton has also been known to be environment dependent. Moreover, the predictive accuracy of CHEMTAX is determined by the pigment ratios utilized in the reference matrix, and it has been suggested that CHEMTAX should be calibrated to the assemblages from which samples will be taken (Mackey et al. 1996). To summarize, this study highlights both the advantages and limitations of interpreting molecular and pigment data and suggests that multiple methods be applied to accurately characterize the spatial and temporal variations in the diversity and community structure of phytoplankton.

## Materials and methods

### Sampling

Two cruises were carried out in the PRE in July and November 2020 (Fig. 1). Water samples were collected from 18 sites. At most sites, water samples at three depth layers, i.e., the surface (5 m), the middle (half of the water depths, ranging from 3 to 15 m), and the bottom (5 m above the seafloor), were collected using Niskin bottles mounted on a rosette sampler, which was equipped with a conductivity-temperature-depth (CTD) sensor (Sea-Bird SBE 11plus) and an SBE43 dissolved oxygen (DO) sensor (SeaBird, Bellevue, WA, USA). Subsamples were filtered through 0.45 μm cellulose acetate fiber membranes and the filtrate was stored at − 20 °C until measurements of nutrients.

### Determination of physicochemical variables

Water temperature, salinity, pH, and DO concentration were determined in situ using the CTD and YSI sensors. The frozen subsamples were thawed at room temperature in laboratory and the concentrations of NO_2_^−^, NO_3_^−^, NH_4_^+^, SRP and DSi were measured using a continuous flow analyzer (Seal AA3, Norderstedt, Germany), with analytical precisions at 0.03 μmol/L, 0.03 μmol/L, 0.03 μmol/L, 0.02 μmol/L, and 0.1 μmol/L, respectively.

### Pigment analysis

To measure the Chl-*a* content and phytoplankton structure, 0.5–1 L of subsamples were prefiltered on board using a 200-µm mesh, then filtered onto 0.7-μm GF/F filters (47 mm in diameter; Whatman, Little Chalfont, Buckinghamshire, UK) under low light and vacuum pressure (< 0.03 MPa) and immediately frozen in liquid nitrogen. Pigment analysis was performed using HPLC (Dionex UltiMate 3000 LC system, Thermo Scientific) (Zhong et al. 2020b). Thirteen diagnostic pigments were used to estimate the relative contributions of five eukaryotic phytoplankton groups [dinoflagellates, diatoms, chlorophytes (including prasinophytes), cryptophytes, haptophytes (a combination of haptophytes_8 and haptophytes_6)] and two prokaryotic groups (*Prochlorococcus* and *Synechococcus*) to the total Chl-*a* using the CHEMTAX program (ver. 1.95) (Mackey et al. 1996). The initial input matrix of ratios of diagnostic pigments to total Chl-*a* followed a previous study of the northern South China Sea (Wang et al. 2015), which included a number of samples from the Pearl River Estuary.

### DNA extraction and high throughput sequencing

The prefiltered subsamples (0.3–0.5 L) for molecular analysis were filtered onto 0.2-μm-pore-sized (47 mm in diameter) polycarbonate membrane (Millipore, Carrigtwohill, Cork, Ireland) under low vacuum. The filters with biomass were put into 2-mL cryovials and immediately stored in liquid nitrogen. DNA extraction was conducted using a FastDNA Spin Kit (MP Biomedical, Santa Anna, CA, USA) according to the manufacturer’s instructions. Eventually, a total of 52 water (18 surface, 16 middle and 18 bottom) samples were subjected to high throughput sequencing of both nuclear 18S and plastid 16S rRNA genes.

The V4 region of 18S rRNA genes was PCR amplified using the primer set TAReuk454FWD1 (5′-CCAGCASCYGCGGTAATTCC-3′) and TAReukREV3 (5′-ACTTTCGTTCTTGATYRA-3′) (Stoeck et al. 2010). The highly variable V4–V5 regions of plastid 16S rRNA genes were amplified using the primers 515F (5′-GTGYCAGCMGCCGCGGTAA-3′) and 926R (5′-CCGYCAATTYMTTTRAGTTT-3′), which were known also to target the 16S rRNA genes of bacteria including cyanobacteria (Mcnichol et al. 2021; Needham and Fuhrman 2016). The PCR reaction solution of each tube (30 μL) contained 1 μL of each primer (10 μmol/L), and 25 μL 2 × *Taq* PCR Master Mix, and 2.5 μL DNA template. The PCR program ran under the following conditions: 95 °C for 5 min, then 7 cycles of 95 °C for 45 s, 65 °C for 1 min (decreasing at 2 °C /cycle), and 72 °C for 90 s, followed by 30 cycles of 95 °C for 45 s, 50 °C for 30 s, and 72 °C for 95 s, with a final extension step for 10 min at 72 °C. The libraries of 16S and 18S rRNA amplicons were sequenced on the Illumina NovaSeq PE250 platform at a commercial company (Novogene, China).

### Processing of sequencing data

The sequence data were processed using QIIME2 (Bolyen et al. 2019). ASVs were generated by trimming the raw amplicon sequences of primers using *cutadapt* (Martin 2011) and inputting them into the DADA2 pipeline (ver. 1.8) (Callahan et al. 2016). Reads were filtered with the following parameters: truncLen and trimLen = c(0, 0), truncQ = 2, maxEE = 2, and then the forward and reverse reads were merged using the default parameters (minOverlap = 12, maxMismatch = 0). Chimeras were removed using the *removeBimeraDenovo* command.

For plastid 16S rRNA genes, taxonomy was assigned using the classifier tool implemented in QIIME2 against the PhytoREF database (Decelle et al. 2015). Considering that evolutionary rates of plastid genes were significantly slower than nuclear ones (Wolfe et al. 1987), and the number of plastid 16S reads (on average 10,587 reads per sample) obtained was much lower than that of nuclear 18S (on average 30,365 reads per sample), it was decided to define 16S sequence diversity at a finer resolution by clustering the reads into ASVs. The taxonomic identity of these ASVs was also examined using BLASTn searches against the plastid database in NCBI (https://ftp.ncbi.nlm.nih.gov/refseq/release/plastid).

For 18S rRNA gene sequences, the qualified reads were clustered into OTUs at 97% identity to minimize inflation of OTU richness due to intragenomic polymorphisms, as suggested by Zou et al. (2021). The obtained OTUs were classified using the Protist Ribosomal Reference database (PR2, ver. 4.14) (Guillou et al. 2012) and SILVA (ver. 138) (Quast et al. 2012). For diversity and community structure of eukaryotic phytoplankton communities, the macroalgae (Rhodophyta, Streptophyta and Ulvophyceae) and non-pigmented dinoflagellates, Cercozoa, Ciliophora, Mesomycetozoa, Radiolaria and Fungi were discarded, and only the OTUs of the photosynthetic groups, i.e., pigmented dinophytes (including Gymnodiniales, Peridiniales, Gonyaulacales, Suessiales, Prorocentrale and Dinophysiales), Bacillariophyta, Chlorophyta, Cryptophyta, Haptophyta, and Ochrophyta were retained for subsequent analyses. The reads generated from MiSeq sequencing of 18S and plastid 16S rRNA genes have been deposited in the NCBI database (accession numbers: PRJNA904229 and PRJNA904929).

### Quantitative real-time PCR (qPCR)

The qPCR assays were performed as previously described (Gong et al. 2013), with some modifications. Since the primers for plastid 16S rRNA genes also target heterotrophic bacteria, another set of primers was selected that specifically targeted the plastid 23S rRNA gene of eukaryotic algae (Kang et al. 2018). These two plastid genes are thought to have identical copy numbers in a chloroplastid genome. The plasmid standards (18S rRNA gene of *Thalassiosira* sp. and plastid 23S rRNA gene of *Synechococcus*) were serially diluted in eight tenfold dilutions. The PCR reaction mixture (20 µL) contained 10 µL Master, 1 µL each primer, 2 µL DNA template and 6 µL double-distilled water. The primer sets 345F (5′-AAGGAAGGCAGCAGGCG-3′) and 499R (5′-CACCAGACTTGCCCTCYAAT-3′) (Zhu et al. 2005), and P23MISQF1 (5′-GGACARWAAGACCCTATGMAG-3′) and P23MISQR1 (5′-AGATYAGCCTGTTATCCCT-3′) (Kang et al. 2018) were used for amplifying 18S and 23S rRNA gene abundances of phytoplankton, respectively. The qPCR assay was based on the fluorescence intensity of the SYBR Green dye, and reactions for each sample were carried out in a Roche LightCycler 96 System (Roche Diagnostics, Mannheim, Germany). The cycling conditions were programmed for 18S rRNA genes as follows: an initial denaturation step at 95 °C for 7 min, followed by 40 cycles at 95 °C for 15 s, 60 °C for 60 s and 77 °C for 25 s. A dissociation curve was examined at 95 °C for 15 s, 60 °C for 60 s and 97 °C for 1 s, to ensure that the target sequences being specifically amplified. The thermal cycling for amplifying plastid 23S rRNA genes consisted of 6 min at 94 °C, followed by 40 cycles at 94 °C for 10 s, 55 °C for 20 s, with a final step at 72 °C for 20 s. The dissociation curve was verified at 95 °C for 10 s, 65 °C for 60 s and 97 °C for 1 s. The ‘absolute abundance’ (i.e., rRNA gene copy number per liter of seawater) of an individual taxon of microbial eukaryotes was calculated as follows:

$$ {\text{Absolute gene abundance of a taxon}} = {\text{CN}}_{{{\text{total}}}} \times {\text{ RP}}. $$

where CN_total_ was the total copy number of 18S (or plastid 16S) rRNA genes determined using qPCR for the whole community; and *RP* represented the relative proportion of the taxon revealed by high throughput sequencing of 18S (or plastid 16S) rRNA genes.

### Statistical analysis

The vertical profiles of environmental factors, rRNA gene abundances, concentrations of nutrients, major phytoplankton groups in pigments, and alpha diversity estimators (OTU or ASV richness, Shannon, Simpson and Chao1) were generated using Ocean Data View (Brown 1998). Alpha diversity indices were computed using *vegan* in R (ver. 4.1.3) (Dixon 2003).

Regression analysis was conducted to explore the correlations between the diversity indices of pigmented eukaryotes and environmental variables using the function *stats* in R. Pearson correlations were performed to test the hypothesis that the relative abundance of a major microalgal group in the phytoplankton community showed a similar variational pattern in applications of all three methods (nuclear 18S rRNA gene, plastid 16S rRNA gene and pigment analysis). Non-metric multidimensional scaling (NMDS) was based on Bray–Curtis dissimilarities of the phytoplankton community structure and performed using the package *vegan* in R. Analysis of similarity (ANOSIM) was conducted to statistically test the hypothesis that the community structure of phytoplankton based on variations in the five major eukaryotic groups (diatoms, dinoflagellates, chlorophytes, haptophytes and cryptophytes) was independent of the three methodologies. Principal components analysis (PCA) was performed to visualize the differences in phytoplankton community structure generated using the three methods. The relationships between community structure and environmental factors were explored using canonical correspondence analysis (CCA). All statistical analyses were carried out using the packages in R (ver. 4.1.3).

## Supplementary Information

Below is the link to the electronic supplementary material.

Supplementary file1 (PDF 2324 KB)

## Data Availability

The data that support the findings of this study are included in this published article (and its supplementary information file).

## References

[CR1] Abaychi J, Riley J (1979). The determination of phytoplankton pigments by high-performance liquid chromatography. Anal Chim Acta.

[CR2] Belevich T, Milyutina I (2022). Species diversity of phototrophic picoplankton in the Kara and Laptev Seas. Microbiology.

[CR3] Bennke CM, Pollehne F, Müller A, Hansen R, Kreikemeyer B, Labrenz M (2018). The distribution of phytoplankton in the Baltic Sea assessed by a prokaryotic 16S rRNA gene primer system. J Plankton Res.

[CR4] Bolyen E, Rideout JR, Dillon MR, Bokulich NA, Abnet CC, Al-Ghalith GA, Alexander H, Alm EJ, Arumugam M, Asnicar F, Bai Y, Bisanz JE, Bittinger K, Brejnrod A, Brislawn CJ, Brown CT, Callahan BJ, Caraballo-Rodriguez AM, Chase J, Cope EK (2019). Reproducible, interactive, scalable and extensible microbiome data science using QIIME 2. Nat Biotechnol.

[CR5] Brown M (1998). Ocean data view 4.0. Oceanography.

[CR6] Callahan BJ, Mcmurdie PJ, Rosen MJ, Han AW, Johnson A, Holmes SP (2016). DADA2: high-resolution sample inference from Illumina amplicon data. Nat Methods.

[CR7] Chai C, Jiang T, Cen J, Ge W, Lu S (2016). Phytoplankton pigments and functional community structure in relation to environmental factors in the Pearl River Estuary. Oceanologia.

[CR8] Chen H, Li DH, Jiang AJ, Li XG, Wu SJ, Chen JW, Qu MJ, Qi XQ, Dai J, Zhao R, Zhang WJ, Liu SS, Wu LF (2022). Metagenomic analysis reveals wide distribution of phototrophic bacteria in hydrothermal vents on the ultraslow-spreading Southwest Indian Ridge. Mar Life Sci Technol.

[CR9] Chen ZF, Zhang QC, Kong FZ, Liu Y, Zhao Y, Zhou ZX, Geng HX, Dai L, Zhou MJ, Yu RC (2019). Resolving phytoplankton taxa based on high-throughput sequencing during brown tides in the Bohai Sea, China. Harmful Algae.

[CR10] Claustre H, Hooker SB, Van Heukelem L, Berthon J-F, Barlow R, Ras J, Sessions H, Targa C, Thomas CS, van der Linde D (2004). An intercomparison of HPLC phytoplankton pigment methods using in situ samples: application to remote sensing and database activities. Mar Chem.

[CR11] Decelle J, Romac S, Stern RF, el Bendif M, Zingone A, Audic S, Guiry MD, Guillou L, Tessier D, Le Gall F, Gourvil P, Dos Santos AL, Probert I, Vaulot D, de Vargas C, Christen R (2015). PhytoREF: a reference database of the plastidial 16S rRNA gene of photosynthetic eukaryotes with curated taxonomy. Mol Ecol Resour.

[CR12] Dixon P (2003). VEGAN, a package of R functions for community ecology. J Veg Sci.

[CR02] Dong Y, Cui L, Cao R, Cen J, Zou J, Zhou X, Lu S (2020) Ecological characteristics and teratogenic retinal determination of Cochlodinium geminatum blooms in Pearl River Estuary, South China. Ecotoxicol Environ Saf 191:110226 10.1016/j.ecoenv.2020.110226 31981955

[CR13] Drouin G, Daoud H, Xia J (2008). Relative rates of synonymous substitutions in the mitochondrial, chloroplast and nuclear genomes of seed plants. Mol Phylogen Evol.

[CR14] Eker-Develi E, Berthon J-F, Canuti E, Slabakova N, Moncheva S, Shtereva G, Dzhurova B (2012). Phytoplankton taxonomy based on CHEMTAX and microscopy in the northwestern Black Sea. J Mar Syst.

[CR15] Everitt D, Wright S, Volkman J, Thomas D, Lindstrom E (1990). Phytoplankton community compositions in the western equatorial Pacific determined from chlorophyll and carotenoid pigment distributions. Deep Sea Res Pt A.

[CR16] Falkowski PG, Katz ME, Knoll AH, Quigg A, Raven JA, Schofield O (2004). The evolution of modern eukaryotic phytoplankton. Science.

[CR17] Field CB, Behrenfeld MJ, Randerson JT, Falkowski P (1998). Primary production of the biosphere: integrating terrestrial and oceanic components. Science.

[CR18] Forsythe ES, Williams AM, Sloan DB (2020). Genome-wide signatures of plastid-nuclear coevolution point to repeated perturbations of plastid proteostasis systems across angiosperms. Plant Cell.

[CR19] Fu R, Gong J (2017). Single cell analysis linking ribosomal (r)DNA and rRNA copy numbers to cell size and growth rate provides insights into molecular protistan ecology. J Eukaryot Microbiol.

[CR20] Gao K, Gao G, Wang Y, Dupont S (2020). Impacts of ocean acidification under multiple stressors on typical organisms and ecological processes. Mar Life Sci Technol.

[CR21] Geider RJ (1987). Light and temperature dependence of the carbon to chlorophyll a ratio in microalgae and cyanobacteria: implications for physiology and growth of phytoplankton. New Phytol.

[CR22] Geider RJ, Platt T, Raven JA (1986). Size dependence of growth and photosynthesis in diatoms: a synthesis. Mar Ecol Prog Ser.

[CR23] Godhe A, Asplund ME, Härnström K, Saravanan V, Tyagi A, Karunasagar I (2008). Quantification of diatom and dinoflagellate biomasses in coastal marine seawater samples by real-time PCR. Appl Environ Microbiol.

[CR24] Gong F, Li G, Wang Y, Liu Q, Huang F, Yin K, Gong J, Dolan J (2020). Spatial shifts in size structure, phylogenetic diversity, community composition and abundance of small eukaryotic plankton in a coastal upwelling area of the northern South China Sea. J Plankton Res.

[CR25] Gong J, Dong J, Liu X, Massana R (2013). Extremely high copy numbers and polymorphisms of the rDNA operon estimated from single cell analysis of oligotrich and peritrich cliates. Protist.

[CR26] Gong W, Hall N, Paerl H, Marchetti A (2020). Phytoplankton composition in a eutrophic estuary: comparison of multiple taxonomic approaches and influence of environmental factors. Environ Microbiol.

[CR27] Guillou L, Bachar D, Audic S, Bass D, Berney C, Bittner L, Boutte C, Burgaud G, de Vargas C, Decelle J, del Campo J, Dolan JR, Dunthorn M, Edvardsen B, Holzmann M, Kooistra WHCF, Lara E, Le Bescot N, Logares R, Mahé F (2012). The Protist Ribosomal Reference database (PR2): a catalog of unicellular eukaryote small sub-unit rRNA sequences with curated taxonomy. Nucl Acids Res.

[CR28] Guo L, Sui Z, Liu Y (2016). Quantitative analysis of dinoflagellates and diatoms community via Miseq sequencing of actin gene and v9 region of 18S rDNA. Sci Rep.

[CR01] Hamilton M, Mascioni M, Hehenberger E, Bachy C, Yung C, Vernet M, Worden AZ (2021) Spatiotemporal variations in Antarctic protistan communities highlight phytoplankton diversity and seasonal dominance by a novel cryptophyte lineage. mBio 12:e02973-21 10.1128/mBio.02973-21 PMC8669470 34903046

[CR29] Hansen PJ, Anderson R, Stoecker DK, Decelle J, Altenburger A, Blossom HE, Drumm K, Mitra A, Flynn KJ (2019) Mixotrophy among freshwater and marine protists. In: Encyclopedia of microbiology, 4th edn. Elsevier, pp 199–210

[CR30] Heidenreich KM, Richardson TL (2020). Photopigment, absorption, and growth responses of marine cryptophytes to varying spectral irradiance. J Phycol.

[CR31] Huang H, Yang J, Huang S, Gu B, Xu D (2021). Spatial distribution of planktonic ciliates in the western Pacific Ocean: along the transect from Shenzhen (China) to Pohnpei (Micronesia). Mar Life Sci Technol.

[CR32] Jia H, Chen M, Su W, Zhang S, Zhao K (2019). Structural characteristics and associated factors influencing phytoplankton abundance and species composition in Huangmaohai Bay, Pearl River Estuary. J Coast Res.

[CR33] Jiang ZY, Wang YS, Cheng H, Sun CC, Wu ML (2015). Variation of phytoplankton community structure from the Pearl River estuary to South China Sea. Ecotoxicology.

[CR34] Kana TM, Geider RJ, Critchley C (1997). Regulation of photosynthetic pigments in micro-algae by multiple environmental factors: a dynamic balance hypothesis. New Phytol.

[CR35] Kang HE, Yoon TH, Yoon S, Kim HJ, Park H, Kang CK, Kim HW (2018). Genomic analysis of red-tide water bloomed with *Heterosigma akashiwo* in Geoje. PeerJ.

[CR36] Kirkham AR, Jardillier LE, Tiganescu A, Pearman J, Zubkov MV, Scanlan DJ (2011). Basin-scale distribution patterns of photosynthetic picoeukaryotes along an Atlantic meridional transect. Environ Microbiol.

[CR37] Lin S (2011). Genomic understanding of dinoflagellates. Res Microbiol.

[CR38] Lin Y, Gifford S, Ducklow H, Schofield O, Cassar N (2019). Towards quantitative microbiome community profiling using internal standards. Appl Environ Microbiol.

[CR03] Liu H, Probert I, Uitz H, Claustre H, Aris-Brosou S, Frada M, Not F, de Vargas C (2009) Extreme diversity in noncalcifying haptophytes explains a major pigment prardox in open oceans. Proc Natl Acad Sci USA 106:12803–12808 10.1073/pnas.0905841106 PMC2722306 19622724

[CR39] Liu Q, Zhao Q, McMinn A, Yang EJ, Jiang Y (2021). Planktonic microbial eukaryotes in polar surface waters: recent advances in high-throughput sequencing. Mar Life Sci Technol.

[CR40] Lu Z, Gan J (2015). Controls of seasonal variability of phytoplankton blooms in the Pearl River Estuary. Deep Sea Res Pt II.

[CR41] Mackey M, Mackey DJ, Higgins HW, Wright SW (1996). CHEMTAX—a program for estimating class abundances from chemical markers: application to HPLC measurements of phytoplankton. Mar Ecol Prog Ser.

[CR42] Maki A, Salmi P, Mikkonen A, Kremp A, Tiirola M (2017). Sample preservation, DNA or RNA extraction and data analysis for high-throughput phytoplankton community sequencing. Front Microbiol.

[CR43] Martin M (2011). Cutadapt removes adapter sequences from high-throughput sequencing reads. Embnet J.

[CR44] McNichol J, Berube PM, Biller SJ, Fuhrman JA (2021) Evaluating and improving SSU rRNA PCR primer coverage for bacteria, archaea, and eukaryotes using metagenomes from global ocean surveys. mSystems 6:e0056521 10.1128/mSystems.00565-21 PMC8269242 34060911

[CR45] Needham DM, Fuhrman JA (2016). Pronounced daily succession of phytoplankton, archaea and bacteria following a spring bloom. Nat Microbiol.

[CR46] Niu L, Luo X, Hu S, Liu F, Cai H, Ren L, Ou S, Zeng D, Yang Q (2020). Impact of anthropogenic forcing on the environmental controls of phytoplankton dynamics between 1974 and 2017 in the Pearl River estuary, China. Ecol Indic.

[CR47] Qiu D, Zhong Y, Chen Y, Tan Y, Song X, Huang L (2019). Short-term phytoplankton dynamics during typhoon season in and near the Pearl River Estuary, South China Sea. J Geophys Res Biogeosci.

[CR48] Quast C, Pruesse E, Yilmaz P, Gerken J, Glckner FO (2012). The SILVA ribosomal RNA gene database project: Improved data processing and web-based tools. Nucl Acids Res.

[CR49] Rii YM, Bidigare RR, Church MJ (2018). Differential responses of eukaryotic phytoplankton to nitrogenous nutrients in the North Pacific Subtropical Gyre. Front Mar Sci.

[CR50] Salmaso N, Boscaini A, Pindo M (2020). Unraveling the diversity of eukaryotic microplankton in a large and deep perialpine lake using a high throughput sequencing approach. Front Microbiol.

[CR51] Sathyendranath S, Cota G, Stuart V, Maass H, Platt T (2001). Remote sensing of phytoplankton pigments: a comparison of empirical and theoretical approaches. Int J Remote Sens.

[CR52] Sathyendranath S, Stuart V, Nair A, Oka K, Nakane T, Bouman H, Forget M-H, Maass H, Platt T (2009). Carbon-to-chlorophyll ratio and growth rate of phytoplankton in the sea. Mar Ecol Prog Ser.

[CR53] Shi XL, Lepere C, Scanlan DJ, Vaulot D (2011). Plastid 16S rRNA gene diversity among eukaryotic picophytoplankton sorted by flow cytometry from the South Pacific Ocean. PLoS ONE.

[CR54] Stoeck T, Bass D, Nebel M, Christen R, Jones MDM, Breiner HW, Richards TA (2010). Multiple marker parallel tag environmental DNA sequencing reveals a highly complex eukaryotic community in marine anoxic water. Mol Ecol.

[CR55] Taylor AH, Geider RJ, Gilbert FJ (1997). Seasonal and latitudinal dependencies of phytoplankton carbon-to-chlorophyll a ratios: results of a modelling study. Mar Ecol Prog Ser.

[CR56] Tragin M, Zingone A, Vaulot D (2018). Comparison of coastal phytoplankton composition estimated from the V4 and V9 regions of the 18S rRNA gene with a focus on photosynthetic groups and especially Chlorophyta. Environ Microbiol.

[CR57] Trefault N, De la Iglesia R, Moreno-Pino M, Lopes Dos Santos A, Gerikas Ribeiro C, Parada-Pozo G, Cristi A, Marie D, Vaulot D (2021). Annual phytoplankton dynamics in coastal waters from Fildes Bay, Western Antarctic Peninsula. Sci Rep.

[CR58] Treusch AH, Demir-Hilton E, Vergin KL, Worden AZ, Carlson CA, Donatz MG, Burton RM, Giovannoni SJ (2012). Phytoplankton distribution patterns in the northwestern Sargasso Sea revealed by small subunit rRNA genes from plastids. ISME J.

[CR59] Veldhuis MJ, Cucci TL, Sieracki ME (1997). Cellular DNA content of marine phytoplankton using two new fluorochromes: taxonomic and ecological implications 1. J Phycol.

[CR60] Wang L, Huang B, Liu X, Xiao W (2015). The modification and optimizing of the CHEMTAX running in the South China Sea. Acta Oceanol Sin.

[CR61] Wang Z, Chi Y, Li T, Song W, Wang Y, Wu T, Zhang G, Liu Y, Ma H, Song W (2022). Biodiversity of freshwater ciliates (Protista, Ciliophora) in the Lake Weishan Wetland, China: the state of the art. Mar Life Sci Technol.

[CR62] Wolfe KH, Li WH, Sharp PM (1987). Rates of nucleotide substitution vary greatly among plant mitochondrial, chloroplast, and nuclear DNAs. Proc Natl Acad Sci USA.

[CR63] Wright S, Jeffrey S, Mantoura R, Llewellyn C, Bjørnland T, Repeta D, Welschmeyer N (1991) Improved HPLC method for the analysis of chlorophylls and carotenoids from marine phytoplankton. Mar Ecol Prog Ser 183–196

[CR64] Wu W, Liu H (2018). Disentangling protist communities identified from DNA and RNA surveys in the Pearl River—South China Sea continuum during the wet and dry seasons. Mol Ecol.

[CR65] Xie ZX, Yan KQ, Kong LF, Gai YB, Jin T, He YB, Wang YY, Chen F, Lin L, Lin ZL, Xu HK, Shao ZZ, Liu SQ, Wang DZ (2022). Metabolic tuning of a stable microbial community in the surface oligotrophic Indian Ocean revealed by integrated meta-omics. Mar Life Sci Technol.

[CR66] Xu S, Liu Y, Fan J, Xiao Y, Qi Z, Lakshmikandan M (2022). Impact of salinity variation and silicate distribution on phytoplankton community composition in Pearl River estuary, China. Ecohydrol Hydrobiol.

[CR67] Yang S, Cui Z, Zhang Y, Jiang T, Yang Q, Sun Y (2019). Photosynthetic pigments in surface sediments in the northwest of the Bohai Sea, China: Potential implications for sediment deposition of brown tides of *Aureococcus anophagefferens* in coastal waters. Ecol Indic.

[CR68] Ye T, Jiang Y, Chen S, Xu Y, Li L, Shin MK, Chen X (2022). The widely reported but poorly studied ciliate family Folliculinidae (Protozoa, Ciliophora, Heterotrichea): a revision with notes on its taxonomy, morphology and phylogenetic relationships. Mar Life Sci Technol.

[CR69] Yeh Y-C, Fuhrman JA (2022). Contrasting diversity patterns of prokaryotes and protists over time and depth at the San-Pedro Ocean Time series. ISME Commun.

[CR04] Yeh YC, Mcnichol J, Needham DM, Fichot EB, Berdjeb L, Fuhrman JA (2021) Comprehensive single-PCR 16S and 18S rRNA community analysis validated with mock communities, and estimation of sequencing bias against 18S. Environ Microbiol 23:3240–3250 10.1111/1462-2920.15553 33938123

[CR70] Zhong Q, Xue B, Noman MA, Wei Y, Liu H, Liu H, Zheng L, Jing H, Sun J (2020). Effect of river plume on phytoplankton community structure in Zhujiang River estuary. J Oceanol Limnol.

[CR71] Zhong Y, Liu X, Xiao W, Laws EA, Chen J, Wang L, Liu S, Zhang F, Huang B (2020). Phytoplankton community patterns in the Taiwan Strait match the characteristics of their realized niches. Prog Oceanogr.

[CR72] Zhu F, Massana R, Not F, Marie D, Vaulot D (2005). Mapping of picoeucaryotes in marine ecosystems with quantitative PCR of the 18S rRNA gene. FEMS Microbiol Ecol.

[CR73] Zou S, Fu R, Zhang Q, Gentekaki E, Gong J (2021). Coupling between ribotypic and phenotypic traits of protists across life-cycle stages and temperatures. Microbiol Spectr.

