# Supplementary material for: Diversity, community structure, and quantity of eukaryotic phytoplankton revealed using 18S rRNA and plastid 16S rRNA genes and pigment markers: a case study of the Pearl River Estuary
Source: Mar Life Sci Technol. 2023 Jul 29;5(3):415–30. doi: 10.1007/s42995-023-00186-x (PMC10449762; doi:10.1007/s42995-023-00186-x)
Supplement: Supplementary file 1 — Supplementary file1 (PDF 2324 KB) [file 42995_2023_186_MOESM1_ESM.pdf]

**Running title:** Characterizing phytoplankton using multiple biomarkers

# Diversity, community structure, and quantity of eukaryotic phytoplankton revealed using 18S rRNA and plastid 16S rRNA genes and pigment markers: A case study of the Pearl River Estuary

**Shumin Xu[1, 2], Guihao Li[1], Cui He[1], Yi Huang[1], Dan Yu[1], Huiwen Deng[1], Zhuyin Tong[3], Yichong Wang[3], Christine Dupuy[4], Bangqin Huang[3], Zhuo Shen[1], Jie Xu[5], Jun Gong[1, 2*]**

[1] School of Marine Sciences, Sun Yat-Sen University (Zhuhai Campus), and Southern Marine Science and Engineering Guangdong Laboratory (Zhuhai), Zhuhai 519000, China

[2] Guangdong Provincial Key Laboratory of Marine Resources and Coastal Engineering, Guangzhou 510006, China

[3] State Key Laboratory of Marine Environmental Science, Xiamen University, Xiamen 361102, China

[4] BIOFEEL, UMRi LIENSs, La Rochelle Université / CNRS, France

[5] Centre for Regional Oceans, Department of Civil and Environmental Engineering, Faculty of Science and Technology, University of Macau, Macau, China

***Correspondence:**

Jun Gong, gongj27@mail.sysu.edu.cn

## Supplementary Figures

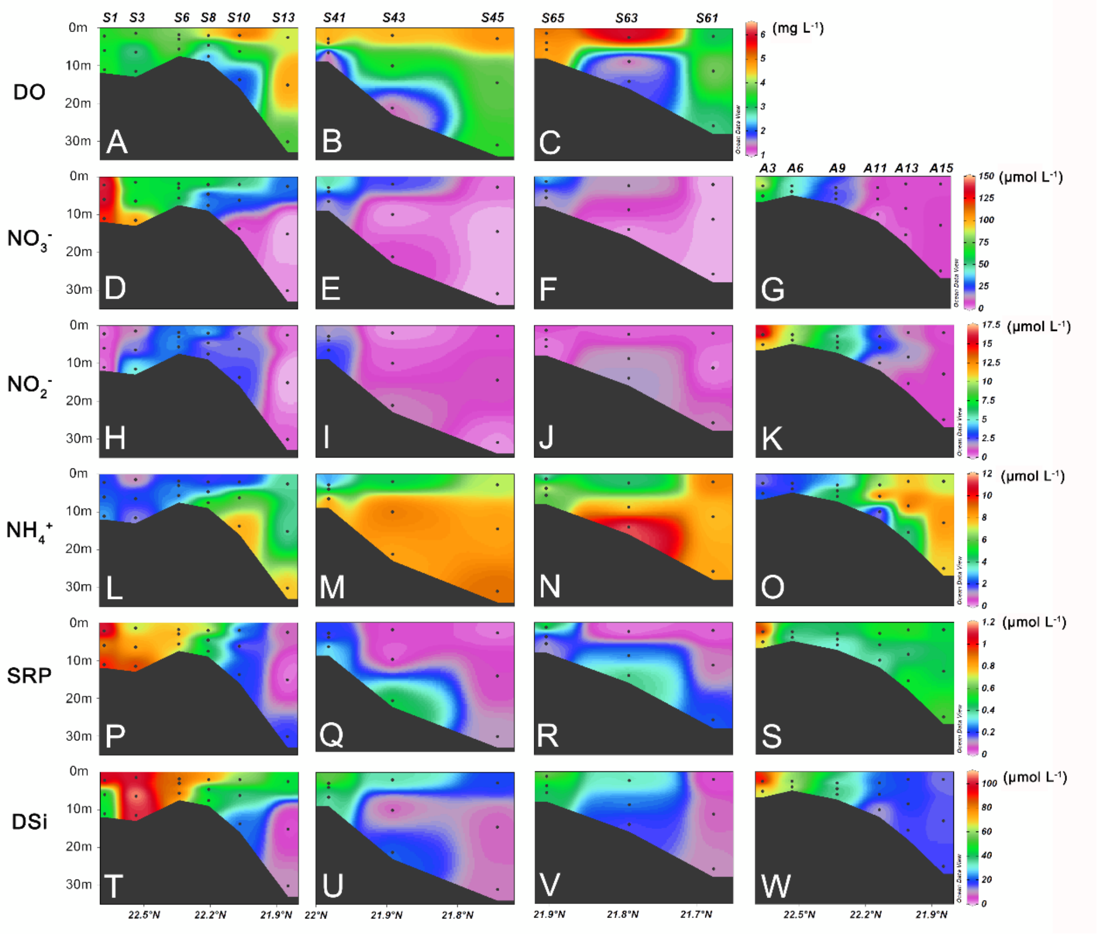


**Fig. S1.** The vertical profiles of concentrations of dissolved oxygen (DO), nitrate ($NO_3^-$), nitrite ($NO_2^-$), ammonia ($NH_4^+$), soluble reactive phosphorate (SRP), and dissolved silicate (DSi) during July (three columns on the left) and November (the rightmost column).

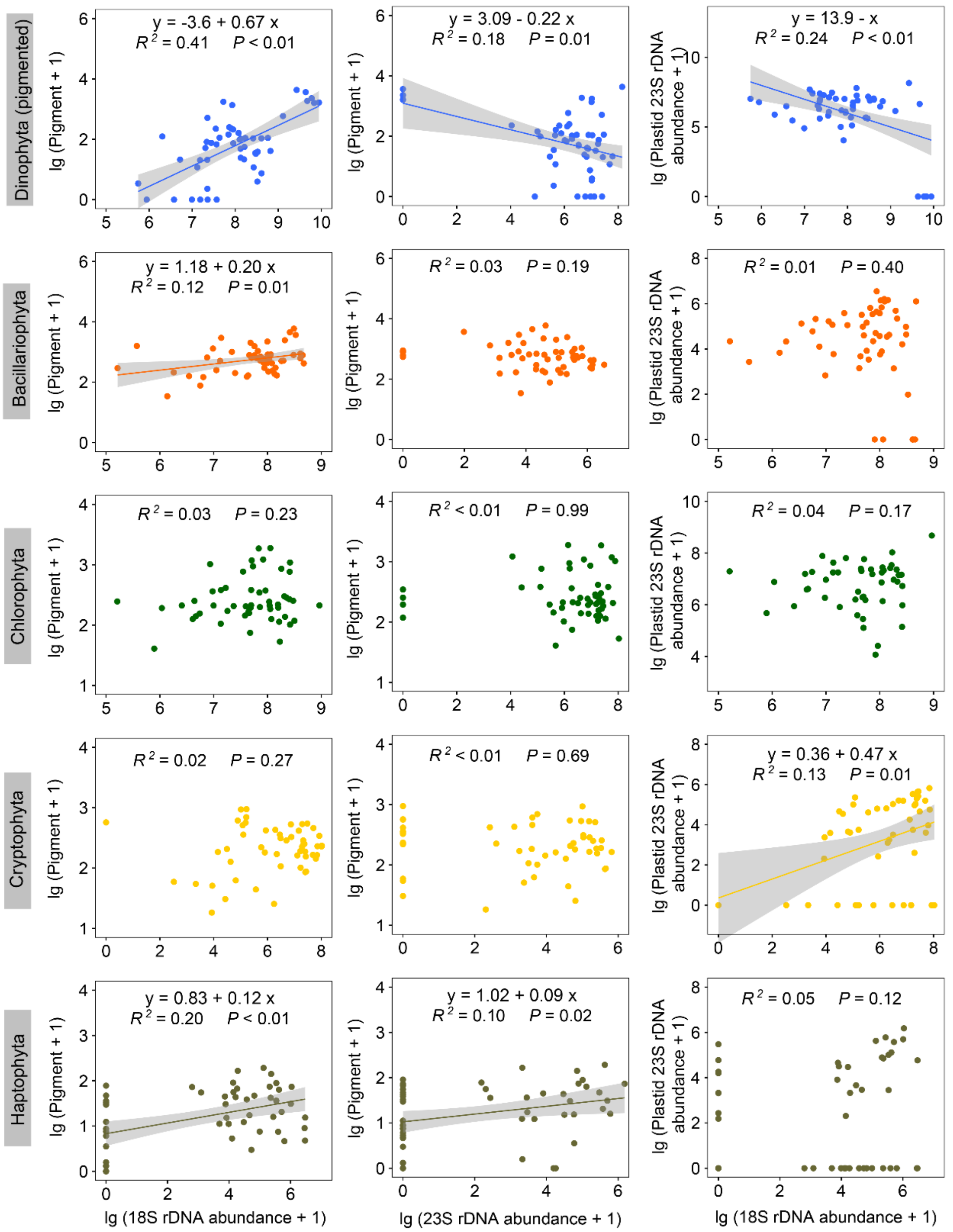


**Fig. S2.** Correlations among 18S rRNA gene abundance (copy number $L^{-1}$), plastid 23S rRNA gene abundance (copy number $L^{-1}$), and pigment content (μg $L^{-1}$) of pigmented dinophytes, chlorophytes, diatoms, cryptophytes, and haptophytes. All gene abundances and pigment contents are log(x+1) transformed.

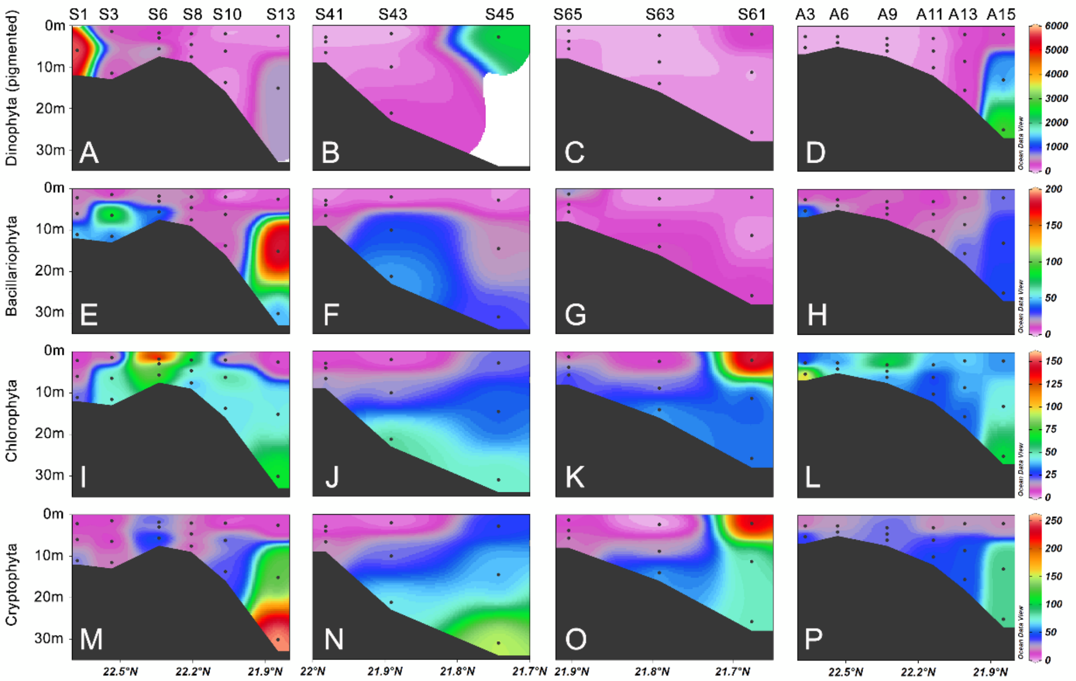


**Fig. S3.** Distribution of ratio of log (18S rRNA gene abundance +1) to chlorophyll *a* in pigmented dinophytes, diatoms, chlorophytes, and cryptophytes. Units of gene abundance and pigment content are copy number $L^{-1}$ and μg $L^{-1}$, respectively.

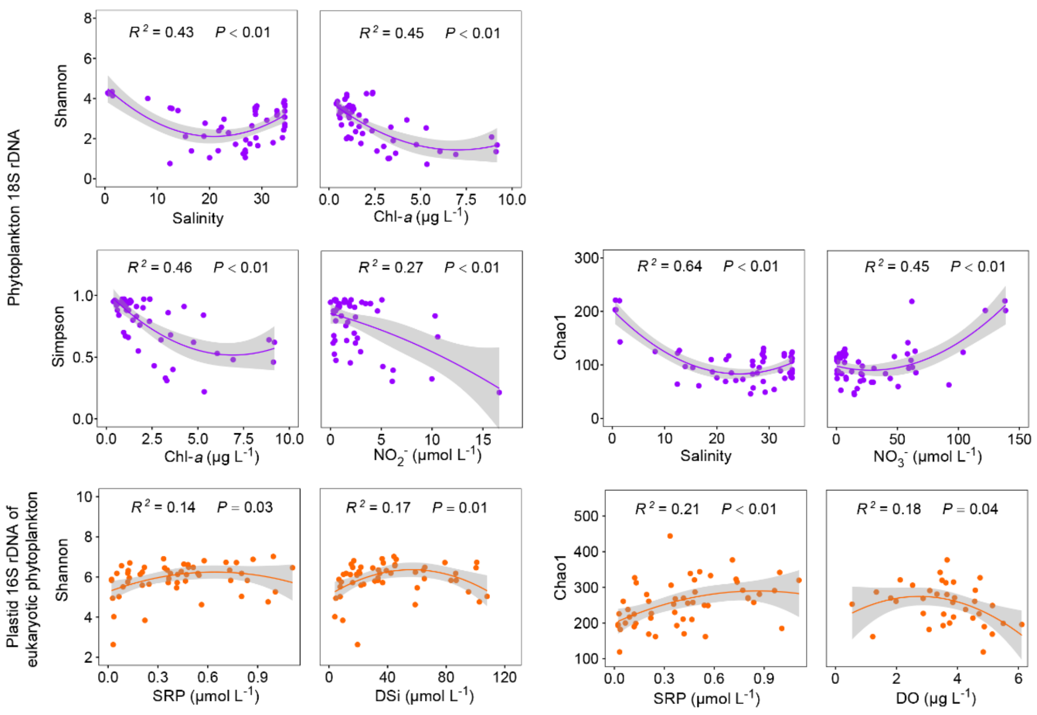


**Fig. S4.** Regression analysis between alpha diversity estimators (Shannon, Chao1 and Simpson indices) of 18S OTUs and 16S ASVs and environmental factors. Only the two most significant environmental factors for each index are shown. Abbreviations: Chl-*a*, chlorophyll *a;* DO, dissolved oxygen; DSi, dissolved silicate; SRP, soluble reactive phosphate.

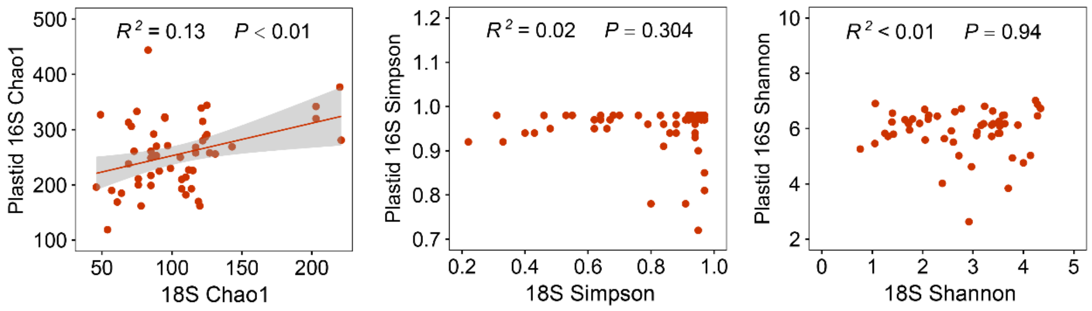


**Fig. S5.** Correlations between of alpha diversity estimators (Chao1, Simpson and Shannon) of 18S and 16S of the total eukaryotic phytoplankton community.

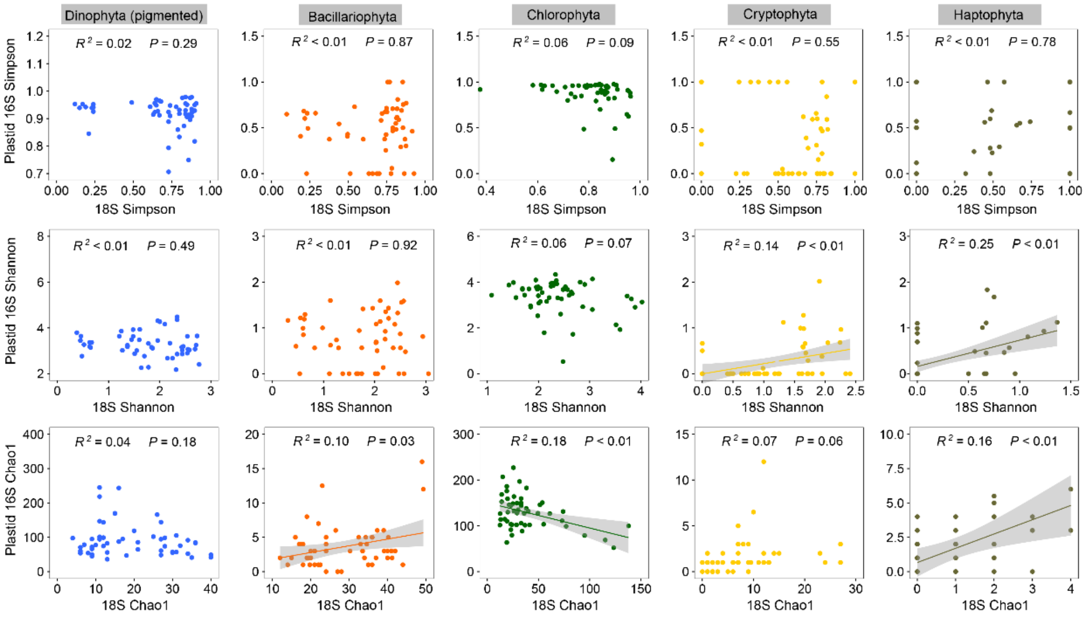


**Fig. S6.** Correlations between of alpha diversity estimators (Chao1, Simpson and Shannon) of 18S and 16S of pigmented dinophytes, diatoms, chlorophytes, cryptophytes and haptophytes.

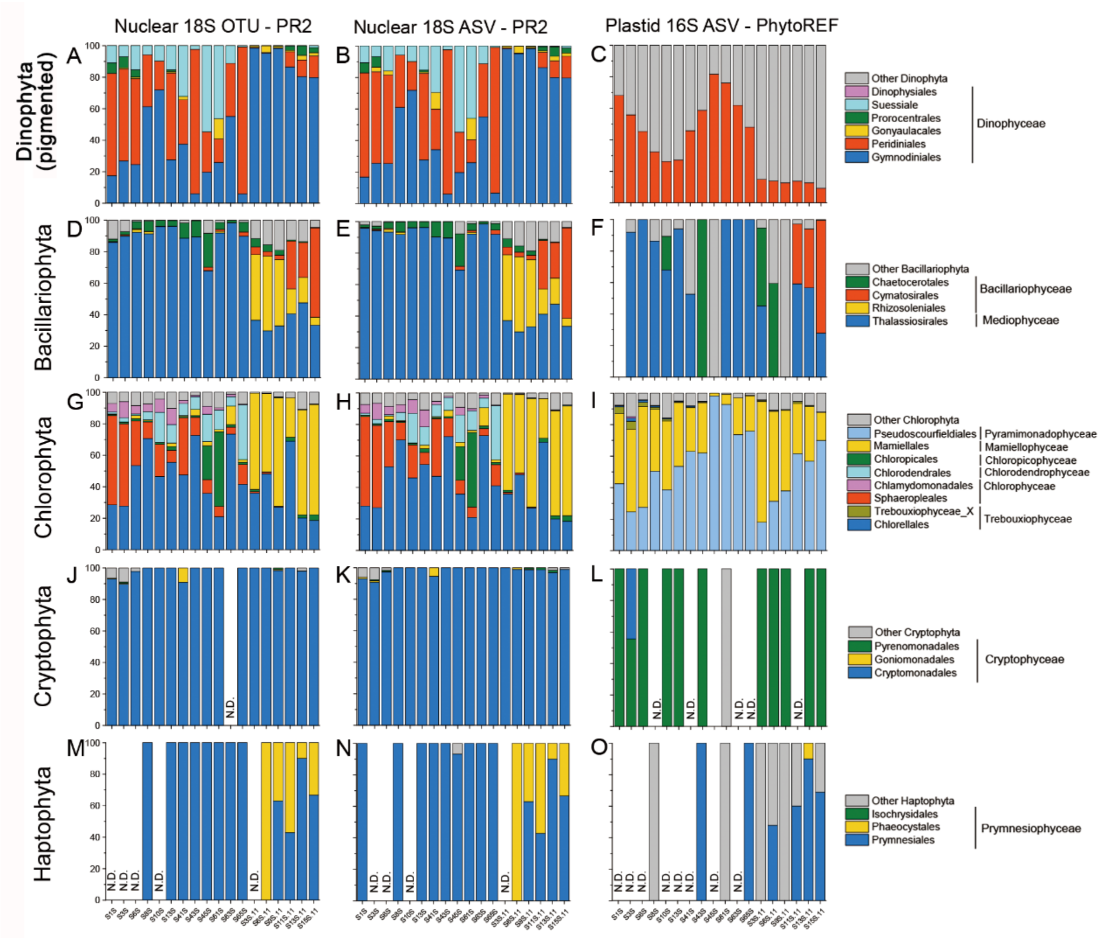


**Fig. S7.** Variations in community composition of eukaryotic phytoplankton in the surface waters. Reads of taxa are binned at order levels. The classification of 18S (left and middle panels) by PR2 yielded almost identical community composition; however the classification of plastid 16S rRNA genes by PhytoRef (right panel) showed different community composition, no matter what resolutions (OTU, defined at a 97% similarity cutoff, and ASV, defined at a 100% similarity cutoff) were applied. N.D., not detected.

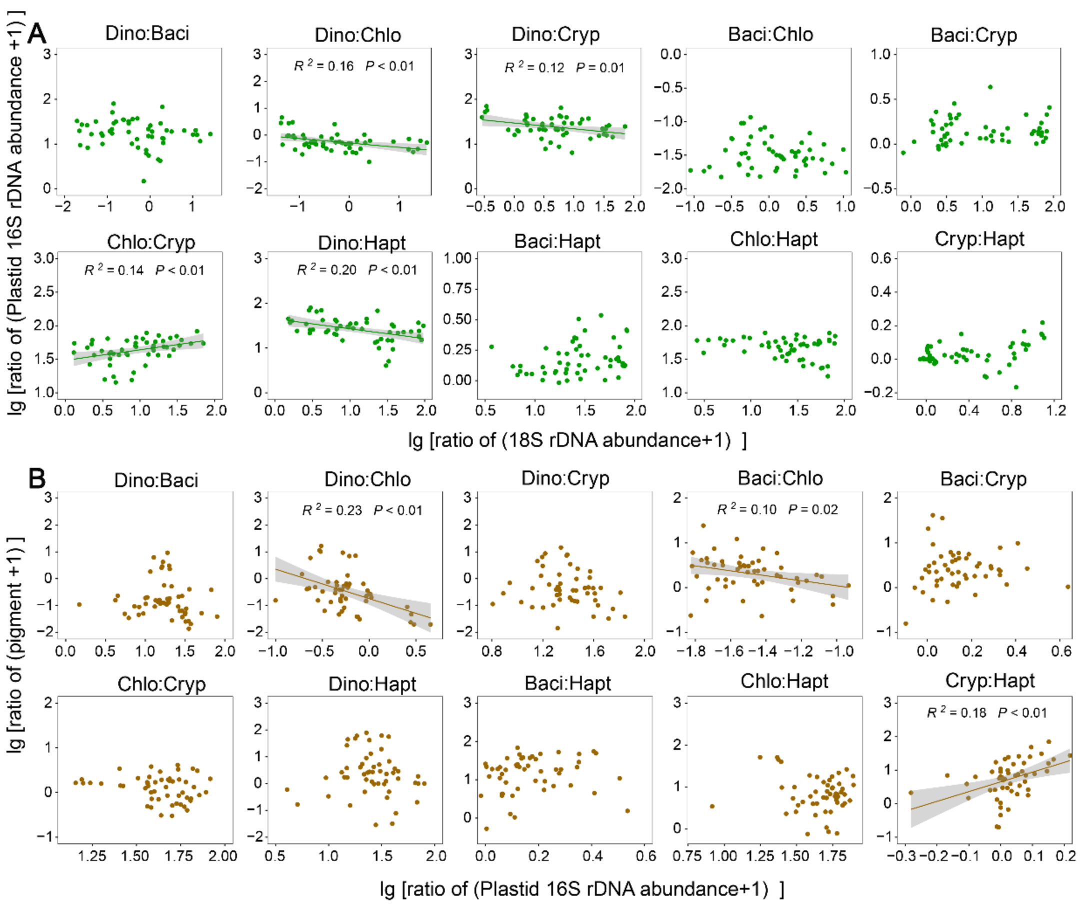


**Fig. S8.** Correlations between ratio of 18S rRNA gene abundance of two microalgal groups and ratio of 16S rRNA gene abundance of those two groups (A), and between 16S rRNA gene abundance of two microalgal groups and pigment contents of the taxon pairs (B). Note that both the gene abundance and pigment content were $\log_{10}(x+1)$ transformed. Abbreviations: Dino, pigmented dinophytes; Baci, Bacillariophyta; Chlo, Chlorophyta; Cryp, Cryptophyta; Hapt, Haptophyta.
